# Microbial Influences on Calcium‐Phosphorus Homeostasis and Metabolic Bone Diseases: A Bidirectional Mendelian Randomisation Study on the Gut–Bone Axis

**DOI:** 10.1111/jcmm.70491

**Published:** 2025-04-01

**Authors:** Yanling Zhou, Yao Yang, Wanbo Zhu, Nikolaos Kourkoumelis, Yingjie Wang, Yuan Chen, Lingxiang Hong, Junjie Wang, Junchen Zhu, Chen Zhu, Xianzuo Zhang

**Affiliations:** ^1^ Department of Orthopedics The First Affiliated Hospital of USTC, Division of Life Sciences and Medicine, University of Science and Technology of China Hefei Anhui China; ^2^ Department of Orthopedics The Second Affiliated Hospital of Anhui University of Chinese Medicine Hefei China; ^3^ Department of Orthopedics Shanghai Sixth People's Hospital Affiliated to Shanghai, Jiao Tong University School of Medicine, Shanghai Jiao Tong University Shanghai P. R. China; ^4^ Department of Medical Physics School of Health Sciences, University of Ioannina Ioannina Greece

**Keywords:** calcium‐phosphorus metabolism, gut microbiota, gut–bone axis, Mendelian randomisation, metabolic bone diseases

## Abstract

Observational studies have shown that the gut microbiota (GM) is associated with bone diseases, particularly calcium‐phosphorus metabolic bone diseases, demonstrating the existence of a gut–bone axis. However, whether these associations are causal effects remains to be determined. This study employed bidirectional two‐sample Mendelian randomisation (MR) using summary data from Genome‐Wide Association Studies (GWAS) of 211 gut microbial taxa and six metabolic bone diseases (osteoporosis, Osteopenia, osteonecrosis, osteomyelitis, hypoparathyroidism and hyperparathyroidism) to explore causal relationships and their directionality. Comprehensive sensitivity analyses were conducted to ensure the robustness of the results, and a false discovery rate‐corrected *p*_FDR_ of < 0.05 was used as a threshold to support strong associations. Additionally, co‐localisation analysis was conducted to consolidate the findings. We identified 35 causal relationships between GM and metabolic bone diseases, with 17 exhibiting positive and 18 negative correlations. Furthermore, reverse MR analysis indicated that osteomyelitis was associated with elevated abundance of two GMs (*p*_FDR_ < 0.05, PP.H4 < 75%). No evidence of horizontal pleiotropy or heterogeneity was observed, and co‐localisation analysis further strengthened the evidence for these causal relationships. The study underscores the critical role of GM in influencing bone health through the gut–bone axis, paving the way for future therapeutic interventions targeting the gut–bone axis and offering new directions for research in bone metabolism and diseases.

## 1 Introduction

The skeleton, as a dynamically remodelling organ, continuously undergoes the closely related processes of bone formation and bone resorption, which are essential for bone metabolism. The homeostasis of bone depends on the dynamic equilibrium of bone metabolism [1]. Metabolic bone diseases, particularly those affected by disturbances in calcium and phosphorus metabolism, represent a significant area of focus in medical research and patient care [2]. Calcium and phosphorus are the primary minerals that constitute bone strength and structure [3]. The balance of bone metabolism is crucial for maintaining the metabolic equilibrium of minerals such as calcium and phosphorus, as well as aspects like bone density and bone strength [4]. Imbalances in bone metabolism can lead to a variety of metabolic bone diseases, such as osteoporosis, osteopenia, osteonecrosis, osteomyelitis, hypoparathyroidism and hyperparathyroidism. Understanding the nuances of calcium‐phosphorus metabolism is, therefore, paramount in addressing these bone diseases [5, 6].

The gut microbiota (GM), a complex ecosystem composed of microorganisms residing in the gastrointestinal tract, plays a crucial role in maintaining the balance of the digestive system, regulating immune responses and synthesising essential nutrients [7]. The GM not only influences local gut health but also impacts systemic processes and may contribute to the development of bone diseases. Increasing evidence suggests a novel and explicit interrelationship between the GM and bone metabolism, termed the ‘gut–bone axis’. The GM indirectly regulates skeletal metabolism by affecting the absorption of calcium and phosphorus, as well as the regulation of bone remodelling [8, 9]. Additionally, interventions such as probiotics can modulate the GM, thereby improving bone mineral density and offering new avenues for the prevention and treatment of bone metabolic diseases [10, 11]. A deeper understanding of the regulatory mechanisms of the gut–bone axis may help uncover broader therapeutic targets for metabolic bone diseases.

Historically, the exploration of the gut–bone axis has predominantly relied on animal models. While these studies have shed light on the potential mechanisms through which GM influences bone health, they do not fully replicate human physiology and disease. Animal models often fail to capture the complexity and diversity of human GM and its interaction with human‐specific genetic and environmental factors [12, 13]. Consequently, translating these findings to human physiology and clinical practice remains challenging. In human studies, most of the research has been limited to cross‐sectional analyses. These studies have primarily focussed on comparing the GM composition in patients with metabolic bone diseases to healthy controls [14, 15]. While such studies provide valuable correlative data, they fall short in establishing a direct causal relationship between specific GM and bone metabolism. The reliance on faecal sample analyses in these studies has further limited the depth of understanding, as it provides only a snapshot of the microbiome and its potential influence on bone health.

In recent years, statistical methods based on Genome‐Wide Association Studies (GWAS) have been developed to estimate correlations and causal relationships between traits. Mendelian randomisation (MR), utilising genetic variants as instrumental variables, has gained significant attention in the medical field for inferring causal relationships between variables [16]. In contrast to observational studies, MR can avoid the influence of confounders. In addition, co‐localisation analysis provides precise information on whether specific single‐nucleotide polymorphisms (SNPs) simultaneously affect multiple traits, aiding in a more comprehensive understanding and interpretation of MR results. As a complement to MR‐Egger, co‐localisation analysis also offers further evidence to support or question the reliability of MR findings. Our study, leveraging GWAS and gene prediction methods, dissects the complex relationship between specific GM and calcium‐phosphorus metabolic bone diseases, aiming to lay the groundwork for innovative and effective strategies in prevention and treatment. This contributes to improved patient prognosis and a deeper understanding of the intricate links between the gut microbiome and overall health.

## 2 Methods

### 2.1 Research Design

**FIGURE 1:**
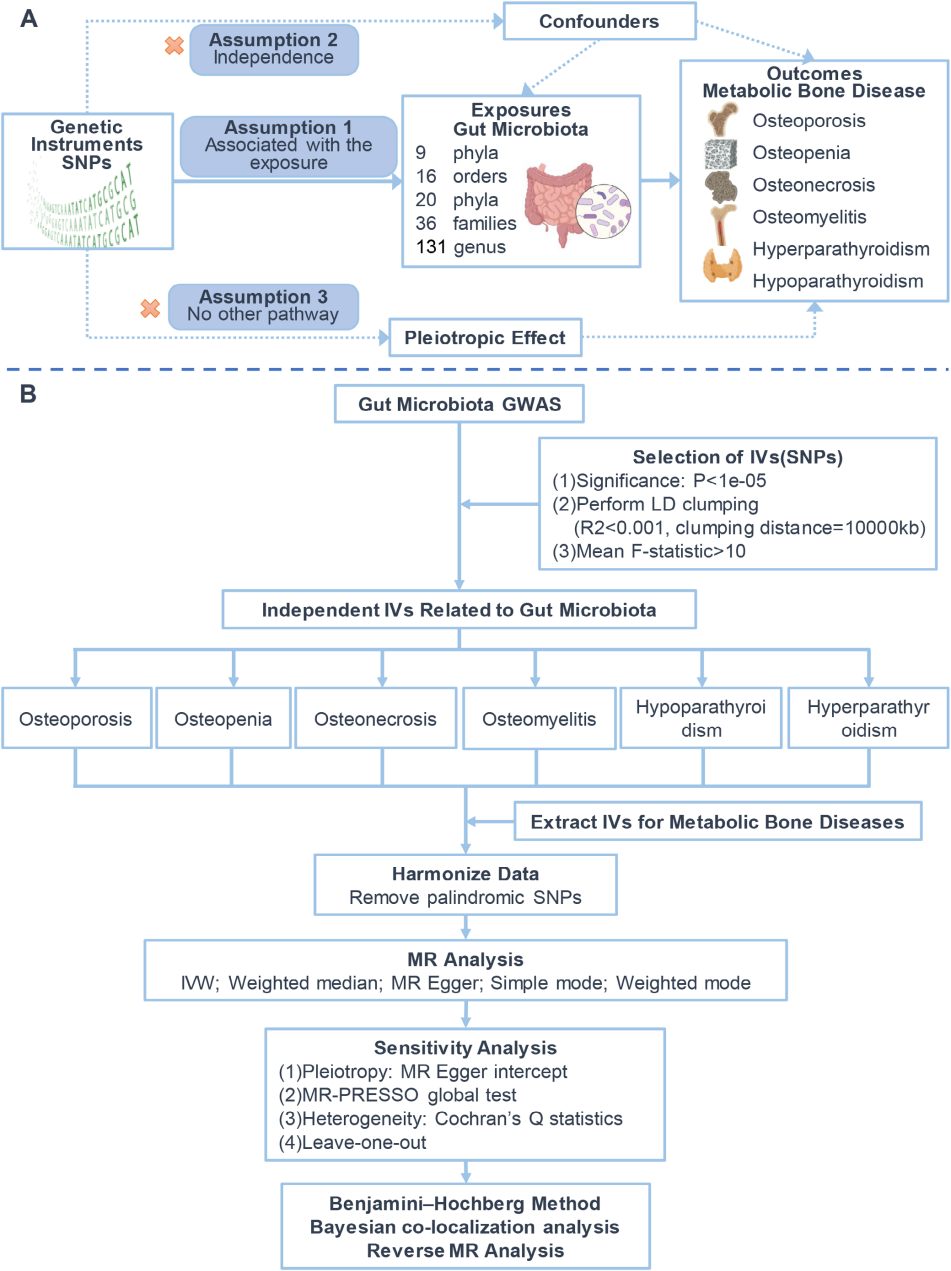
The foundational hypotheses (A) and workflow diagram (B) for Mendelian randomisation (MR) analyses. GWAS, genome‐wide association study; IVs, instrumental variables; IVW, Inverse‐variance weighted; LD, linkage disequilibrium; MR‐PRESSO, Mendelian randomisation pleiotropy RESidual Sum and Outlier; SNPs, single‐nucleotide polymorphisms.

Utilising a bidirectional two‐sample MR framework, this study rigorously evaluates the potential causal effects of the GM on a spectrum of prevalent metabolic bone diseases, namely osteoporosis, osteopenia, osteonecrosis, osteomyelitis, hypoparathyroidism and hyperparathyroidism. The assessment spans multiple taxonomic levels, encompassing phylum, class, order, family and genus. According to strict inclusion and exclusion criteria, SNPs significantly associated with specific gut microbial taxa were selected as instrumental variables (IVs). For results with significant associations, we conducted a series of sensitivity analyses and applied the Benjamini–Hochberg method to correct the false discovery rate (FDR). To determine the direction of causal effects and to investigate the impact of bone metabolic diseases on GM, we performed reverse MR analyses. To consolidate the results of the analyses, we also performed Bayesian co‐localisation analysis. Our research was reported in accordance with the ‘Strengthening the Reporting of Observational Studies in Epidemiology using Mendelian Randomization (STROBE‐MR)’ checklist [17]. The foundational hypotheses and workflow diagram are depicted in Figure 1.

### 2.2 Exposure Data

The comprehensive profiling of the GM in our study is derived from an extensive multi‐ethnic meta‐analysis conducted by the MiBioGen Consortium (https://mibiogen.gcc.rug.nl/) [18]. This significant research coordinated 16S rRNA gene sequencing profiles and genotyping data from a broad cohort of 18,340 individuals drawn from 24 diverse groups, with a predominant representation of European ancestry (*N* = 13,266). A total of 122,110 SNPs were included across 9 phyla, 16 classes, 20 orders, 35 families (with 3 unknown families) and 131 genera (with 15 unknown genera).

### 2.3 Outcome Data

The GWAS summary data for osteoporosis, osteopenia and osteomyelitis were acquired from the UK Biobank of European descent [19]. Among them, there were 7547 cases and 455,386 controls for osteoporosis, 1145 cases and 461,788 controls for osteopenia and 4836 cases and 481,648 controls for osteomyelitis. The summary genetic data for osteonecrosis, hyperparathyroidism and hypoparathyroidism were acquired from FinnGen of European descent [20]. Among them, there were 604 cases and 209,575 controls for osteonecrosis, 2928 cases and 211,123 controls for hyperparathyroidism and 485 cases and 211,123 controls for hypoparathyroidism. Detailed information regarding the data used can be found in Table S1.

### 2.4 Selection of Instrumental Variables

In the process of selecting appropriate genetic instrumental variables (IVs) for our MR analysis, a series of criteria were employed: (i) Relaxing the Significance Threshold: Due to the typically low number of eligible IVs under the genome‐wide significance threshold (*p* < 5 × 10^−8^), a relatively less stringent threshold (*p* < 1 × 10^−5^) was chosen. This approach, based on prior studies [21, 22, 23], was aimed at capturing a potential set of variants enriched for associations and to obtain more comprehensive results. (ii) Ensuring Independence of SNPs: We conducted a clumping procedure (*R* ^2^ < 0.001, clumping distance = 10,000 kb) to exclude variants in strong linkage disequilibrium (LD) and to ensure the independence of each SNP. (iii) Exclusion criteria for SNPs: SNPs with allele frequencies less than 0.01, ambiguous SNPs with inconsistent alleles and palindromic SNPs were excluded. (iv) Phenotype Association Screening: Utilising gene scanning tools [24], we identified all known phenotypes associated with genetic IVs (*p* < 5 × 10^−5^). Any IV associated with other known phenotypes was excluded from subsequent MR analysis. We also referenced the largest GWAS to date, which included dietary habits such as intake of raw vegetables, fresh fruits and oily fish. Genetic IVs associated with these 20 dietary habits were removed. Furthermore, the strength of each instrumental variable was assessed using the F statistic [25]. An *F* value greater than 10 was indicative of robust IVs, signifying minimal bias. IVs with *F* values less than 10 were deemed weak and excluded from the analysis. The formula for the *F* statistic is:

$$F=R^{2}\left(n-k-1\right)/k\left(1-R^{2}\right)$$

where *R* ^2^ represents the proportion of exposure variance explained by IVs, *n* is the sample size, and *k* is the number of IVs. The PhenoScanner database webtool [26] was employed to eliminate potential confounders associated with the instrumental variables, thereby preventing interference in the exposure –outcome relationship.

### 2.5 MR Analysis

Five popular MR methods were used for features containing multiple IVs: inverse‐variance weighted (IVW) test [27], weighted mode [28], MR‐Egger regression [24], weighted median estimator (WME) [29] and Simple mode [30]. The number of taxa tested was corrected for multiple testing using the Benjamini–Hochberg FDR correction, and the statistical significance of the MR effect estimate was defined as FDR < 0.05 [31]. All statistical analyses were performed using the “TwoSampleMR” (version 0.5.6) and “MendelianRandomization” (version 0.6.0) packages in R (version 4.1.2).

### 2.6 Sensitivity Analyses

To validate the causality of these highly probable and possible relationships, we conducted sensitivity analyses. These analyses tested whether the MR assumptions were potentially violated. The MR‐Egger [24] and MR‐Pleiotropy Residual Sum and Outlier (MR‐PRESSO) tests [32] were utilised to assess horizontal pleiotropy and outliers. The MR‐Egger regression intercepts provided an indication of potential horizontal pleiotropy, with *p*‐values greater than 0.05 suggesting no significant horizontal pleiotropy [24]. The MR‐PRESSO test sequentially eliminated outliers based on their *p*‐values, continuing until the global *p*‐value exceeded 0.05, effectively ruling out horizontal polyvalence. SNPs remaining after the removal of pleiotropic outliers were carried forward to the next stage of MR analysis [32]. Cochrane's *Q*‐test [33] was employed to quantify the heterogeneity among IVs. Additionally, leave‐one‐out sensitivity analyses were conducted on individual SNPs to identify those potentially contributing to heterogeneity. Furthermore, to assess the causal relationship between GM and metabolic bone diseases, reverse MR analyses were performed on bacteria found to be causally associated with metabolic bone diseases in the initial MR analyses. The methods and settings for these reverse analyses were consistent with those used in the forward MR. For these analyses, R version 4.2.2 was used, primarily employing the ‘TwoSampleMR’ [16] and ‘MR‐PRESSO’ [34] packages.

### 2.7 Bayesian Co‐Localisation Analysis

To further reinforce the validation of causality and mitigate biases from pleiotropy and LD, we employed Coloc2 (https://github.com/chr1swallace/coloc) [35], an advanced method addressing allelic heterogeneity, for co‐localisation analysis. For each leading SNP in the GWAS database under study, all SNPs within 500 kb upstream and downstream of the leading SNP were retrieved for co‐localisation analysis to analyse Posterior probabilities hypothesis 4 (PP.H4). Co‐localisation analysis evaluates whether genetic loci associated with different traits, such as GM and bone density, share the same genetic signal, thereby reducing biases from pleiotropy and linkage disequilibrium [36]. We consider PP.H4 < 75% as the threshold indicating no significant pleiotropy, as lower PP.H4 values suggest that the genetic signals of the two traits originate from different loci, thus ruling out pleiotropy‐induced confounding. Conversely, PP.H4 exceeding 75% indicates a strong likelihood of shared genetic causal variants between exposure and outcome, providing evidence for horizontal pleiotropy. This can violate the exclusion restriction assumption and potentially lead to biased reasoning and erroneous conclusions [37].

## 3 Results

In this MR analysis, 2819 SNPs were selected as IVs from 211 bacterial taxa, and all *F*‐statistics for these SNPs surpassed the critical threshold of 10, indicating the robustness of the chosen IVs and excluding any weak instrumental variables from our analysis (Table S2). The culmination of our analysis identified 479 SNPs that were associated with GM across various diseases, with the number of SNPs ranging from 3 to 18 for different diseases (Table S3).

### 3.1 Osteoporosis

**FIGURE 2:**
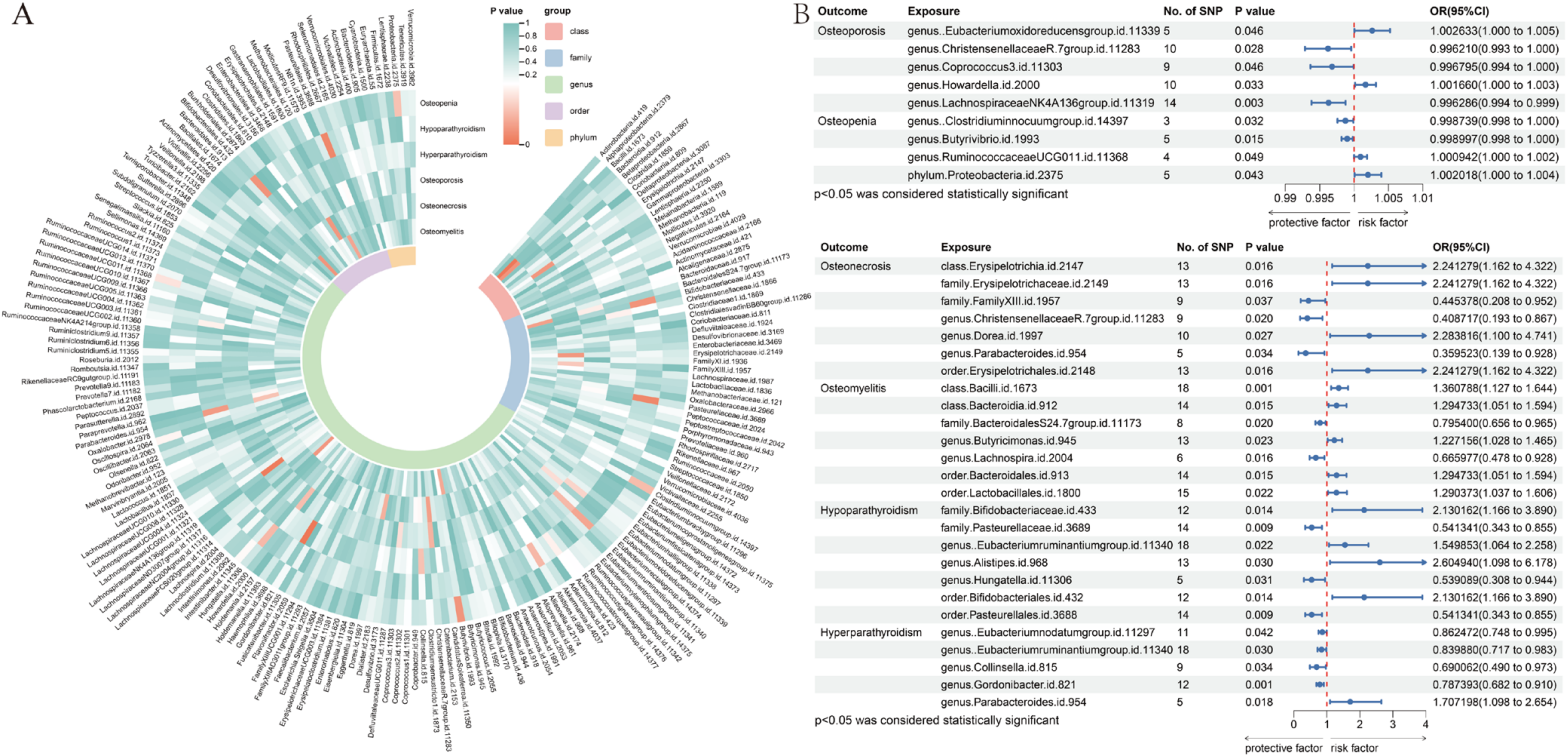
Analysis of the causal relationship between gut microbiota (GM) and metabolic bone diseases. (A) A circular heatmap of the primary results of different classification levels of GM with six metabolic bone diseases. (B) The association between genetically determined GM and the risk of six metabolic bone diseases in the primary results. CI, confidence interval; OR, odds ratios.

In the context of osteoporosis, the IVW analysis revealed that certain microbiota elements were significantly associated with genetic predisposition to the disease (Table S4). Specifically, a higher genetic predisposition was linked with genera like *Christensenellaceae* R.7 (OR = 0.996, 95% CI = 0.993–1.000, *p*_FDR_ = 0.046), *Coprococcus3* (OR = 0.997, 95% CI = 0.994–1.000, *p*_FDR_ = 0.046) and *Lachnospiraceae* NK4A136 (OR = 0.996, 95% CI = 0.994–0.999, *p*_FDR_ = 0.021). On the other hand, the presence of the genus *Eubacterium oxidoreducens* (OR = 1.003, 95% CI = 1.000–1.005, *p*_FDR_ = 0.046) and genus *Howardella* (OR = 1.002, 95% CI = 1.000–1.003, *p*_FDR_ = 0.046) was associated with an increased risk of osteoporosis (Figure 2).

### 3.2 Osteopenia

The IVW analysis for osteopenia, as presented in Table S4, indicated that a higher genetically predicted abundance of genus *Ruminococcaceae* UCG011 (OR = 1.001, 95% CI = 1.000–1.002, *p*_FDR_ = 0.049) and phylum *Proteobacteria* (OR = 1.002, 95% CI = 1.000–1.004, *p*_FDR_ = 0.049) were associated with an elevated risk of osteopenia. In contrast, a higher genetically predicted abundance of genus *Clostridium innocuum* (OR = 0.999, 95% CI = 0.998–1.000, *p*_FDR_ = 0.049) and genus *Butyrivibrio* (OR = 0.999, 95% CI = 0.998–1.000, *p*_FDR_ = 0.049) was associated with a reduced risk of osteopenia. These findings underscore the influence of specific microbiota components on the risk of developing osteopenia (Figure 2).

### 3.3 Osteonecrosis

In the case of osteonecrosis, the IVW analysis (Table S4) demonstrated that higher genetically predicted abundances in class *Erysipelotrichia* (OR = 2.241, 95% CI = 1.162–4.322, *p*_FDR_ = 0.034), family *Erysipelotrichaceae* (OR = 2.241, 95% CI = 1.162–4.322, *p*_FDR_ = 0.034), genus *Dorea* (OR = 2.284, 95% CI = 1.100–4.741, *p*_FDR_ = 0.037) and order *Erysipelotrichales* (OR = 2.241, 95% CI = 1.162–4.322, *p*_FDR_ = 0.034) were associated with an increased risk of osteonecrosis. Conversely, higher genetically predicted abundances in family *FamilyXIII* (OR = 0.445, 95% CI = 0.208–0.952, *p*_FDR_ = 0.037), genus *Christensenellaceae* R.7 (OR = 0.409, 95% CI = 0.193–0.867, *p*_FDR_ = 0.034) and genus *Parabacteroides* (OR = 0.360, 95% CI = 0.139–0.928, *p*_FDR_ = 0.034) were associated with a reduced risk of osteonecrosis, highlighting the significant impact of specific GM components on this condition (Figure 2).

### 3.4 Osteomyelitis

Our study has identified seven causal relationships between GM and the risk of developing osteomyelitis, as detailed in Table S4. IVW estimates indicated that a higher genetic predisposition for increased abundance of the family *Bacteroidales* S24.7 (OR = 0.795, 95% CI = 0.656–0.965, *p*_FDR_ = 0.023) and genus *Lachnospira* (OR = 0.666, 95% CI = 0.478–0.928, *p*_FDR_ = 0.023) was associated with a reduced risk of osteomyelitis. Conversely, an increased abundance of class *Bacilli* (OR = 1.361, 95% CI = 1.127–1.644, *p*_FDR_ = 0.011), class *Bacteroidia* (OR = 1.295, 95% CI = 1.051–1.594, *p*_FDR_ = 0.023), genus *Butyricimonas* (OR = 1.227, 95% CI = 1.028–1.465, *p*_FDR_ = 0.023), order *Bacteroidales* (OR = 1.295, 95% CI = 1.051–1.5942, *p*_FDR_ = 0.023) and order *Lactobacillales* (OR = 1.295, 95% CI = 1.051–1.594, *p*_FDR_ = 0.023) were associated with an increased risk of osteomyelitis (Figure 2).

### 3.5 Hypoparathyroidism

The IVW analysis for hypoparathyroidism (Table S4) showed that a higher genetically predicted abundance of family *Bifidobacteriaceae* (OR = 2.130, 95% CI = 1.166–3.890, *p*_FDR_ = 0.024), genus *Eubacterium ruminantium* (OR = 1.550, 95% CI = 1.064–2.258, *p*_FDR_ = 0.031), genus *Alistipes* (OR = 2.605, 95% CI = 1.098–6.178, *p*_FDR_ = 0.031) and order *Bifidobacteriales* (OR = 2.130, 95% CI = 1.166–3.890, *p*_FDR_ = 0.024) were associated with an elevated risk of hypoparathyroidism. In contrast, higher genetically predicted abundances of family *Pasteurellaceae* (OR = 0.541, 95% CI = 0.343–0.855, *p*_FDR_ = 0.024), genus *Hungatella* (OR = 0.539, 95% CI = 0.308–0.944, *p*_FDR_ = 0.031) and order *Pasteurellales* (OR = 0.541, 95% CI = 0.343–0.855, *p*_FDR_ = 0.024) were associated with a reduced risk of hypoparathyroidism. These findings emphasise the potential influence of specific GM components on the development of hypoparathyroidism (Figure 2).

### 3.6 Hyperparathyroidism

For hyperparathyroidism, the IVW analysis (Table S4) revealed that a higher genetically predicted abundance of the genus *Parabacteroides* (OR = 1.707, 95% CI = 1.098–2.654, *p*_FDR_ = 0.042) was linked with an increased risk. On the contrary, the presence of genera such as *Eubacterium nodatum* (OR = 0.862, 95% CI = 0.748–0.995, *p*_FDR_ = 0.042), *Eubacterium ruminantium* (OR = 0.840, 95% CI = 0.717–0.983, *p*_FDR_ = 0.042), *Collinsella* (OR = 0.690, 95% CI = 0.490–0.973, *p*_FDR_ = 0.042) and *Gordonibacter* (OR = 0.787, 95% CI = 0.682–0.910, *p*_FDR_ = 0.006) were associated with a reduced risk of hyperparathyroidism, underscoring the potential role of specific GM in modulating the risk (Figure 2).

### 3.7 Sensitivity Analysis and Reverse MR Analysis

**FIGURE 3:**
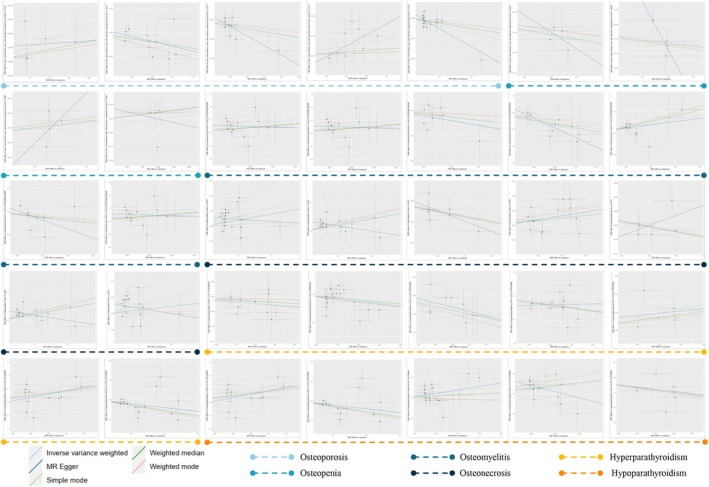
Scatter plots for causal effects of gut microbiota on six metabolic bone diseases. Further details are provided in the Supporting Information.

**FIGURE 4:**
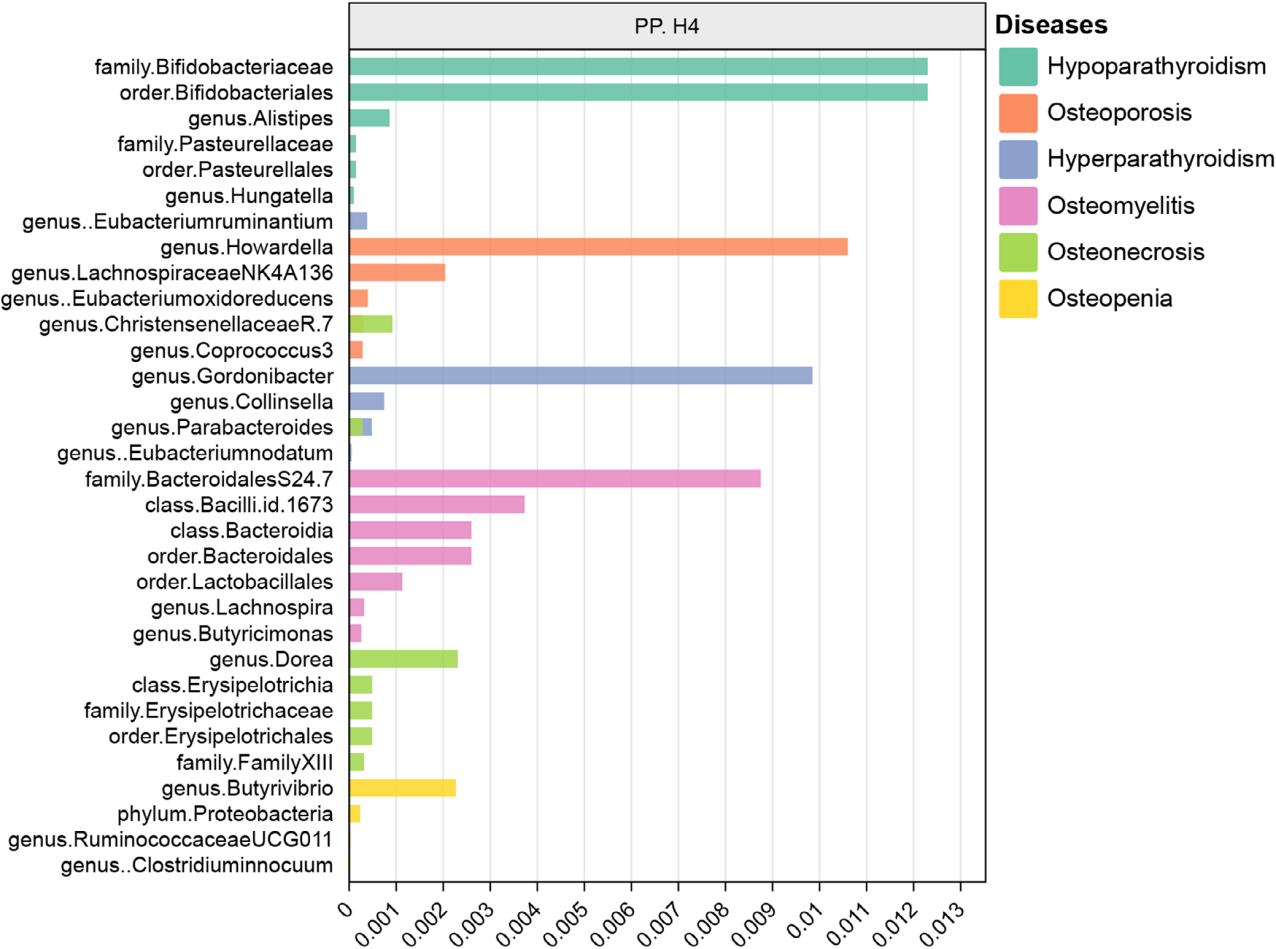
The result of co‐localisation analysis in PP.H4.

A series of sensitivity analyses were conducted to evaluate the heterogeneity and horizontal pleiotropy of the instrumental variables (IVs) selected for the study. Evidence of horizontal pleiotropy or outliers was not observed in the MR‐Egger and MR‐PRESSO tests (*p* > 0.05) (Table 1). In addition, Cochrane's *Q* test revealed no significant heterogeneity among the results (*p* > 0.05) (Table 1). Analysis using the leave‐one‐out method showed no influential outliers among the SNPs, as presented in Supporting Information, Figures S1–S6. Detailed scatter plots for each MR method analysis are provided in Supporting Information, Figures S7–S12 (Figure 3). The results of the Bayesian co‐localisation analysis showed that the PP.H4 for all 35 causal relationships was less than 0.75, strongly suggesting that there are no shared loci between GM with significant causality and metabolic bone disease that are not affected by pleiotropy (Figure 4, Table S5). We plotted LocusZoom plots to visualise these co‐localisations in Supporting Information, Figure S13.

**TABLE 1:** Sensitivity analysis results of causal effects between gut microbiota and six metabolic bone diseases risk (*p* < 5 × 10^−5^).

Outcome	Exposure	*N*	Heterogeneity test		Pleiotropy test		MR‐PRESSO global test
			Cochran *Q*‐value	*p*	Intercept	*p*	*p*
Osteoporosis	Genus *Eubacteriumoxidoreducens*	5	4.423	0.352	0	0.673	0.436
	Genus *Christensenellaceae R.7*	10	10.056	0.346	0	0.556	0.344
	Genus *Coprococcus3*	9	3.191	0.922	0	0.41	0.931
	Genus *Howardella*	10	5.752	0.765	−0.001	0.289	0.783
	Genus *LachnospiraceaeNK4A136*	14	11.362	0.581	0	0.086	0.509
Osteopenia	Genus *Clostridiuminnocuum*	3	0.441	0.802	0	0.754	0.392
	Genus *Butyrivibrio*	5	2.649	0.618	0.002	0.257	0.671
	Genus *RuminococcaceaeUCG011*	4	2.734	0.434	−0.001	0.625	0.469
	Phylum *Proteobacteria*	5	4.377	0.357	0	0.884	0.428
Osteonecrosis	Class *Erysipelotrichia*	13	8.098	0.777	0.062	0.496	0.802
	Family *Erysipelotrichaceae*	13	8.098	0.777	0.062	0.496	0.782
	Family *FamilyXIII*	9	8.05	0.429	−0.004	0.97	0.423
	Genus *Christensenellaceae R.7*	9	2.362	0.984	0.084	0.339	0.987
	Genus *Dorea*	10	4.4	0.883	0.017	0.808	0.91
	Genus *Parabacteroides*	5	2.336	0.674	0.043	0.879	0.74
	Order *Erysipelotrichales*	13	8.098	0.777	0.062	0.496	0.793
Osteomyelitis	Class *Bacilli*	18	18.37	0.366	0.03	0.125	0.395
	Class *Bacteroidia*	14	8.259	0.826	0.038	0.047	0.78
	Family *BacteroidalesS24.7*	8	4.543	0.716	0.026	0.553	0.719
	Genus *Butyricimonas*	13	11.406	0.494	−0.006	0.839	0.532
	Genus *Lachnospira*	6	4.19	0.522	−0.079	0.261	0.539
	Order *Bacteroidales*	14	8.259	0.826	0.038	0.047	0.784
	Order *Lactobacillales*	15	17.715	0.22	0.034	0.116	0.247
Hypoparathyroidism	Family *Bifidobacteriaceae*	12	15.807	0.148	0.02	0.604	0.157
	Family *Pasteurellaceae*	14	14.338	0.351	−0.006	0.931	0.392
	Genus *Eubacteriumruminantium*	18	16.207	0.509	−0.023	0.717	0.53
	Genus *Alistipes*	13	15.704	0.205	0.206	0.099	0.223
	Genus *Hungatella*	5	3.68	0.451	−0.009	0.974	0.534
	Order *Bifidobacteriales*	12	11.554	0.398	−0.038	0.629	0.475
	Order *Pasteurellales*	14	14.338	0.351	−0.006	0.931	0.408
Hyperparathyroidism	Genus *Eubacteriumnodatum*	11	5.738	0.837	−0.002	0.973	0.871
	Genus *Eubacteriumruminantium*	18	14.318	0.644	−0.001	0.977	0.685
	Genus *Collinsella*	9	6.37	0.606	0.016	0.74	0.599
	Genus *Gordonibacter*	12	10.063	0.525	−0.043	0.386	0.556
	Genus *Parabacteroides*	5	3.317	0.506	0.02	0.883	0.588

**FIGURE 5:**
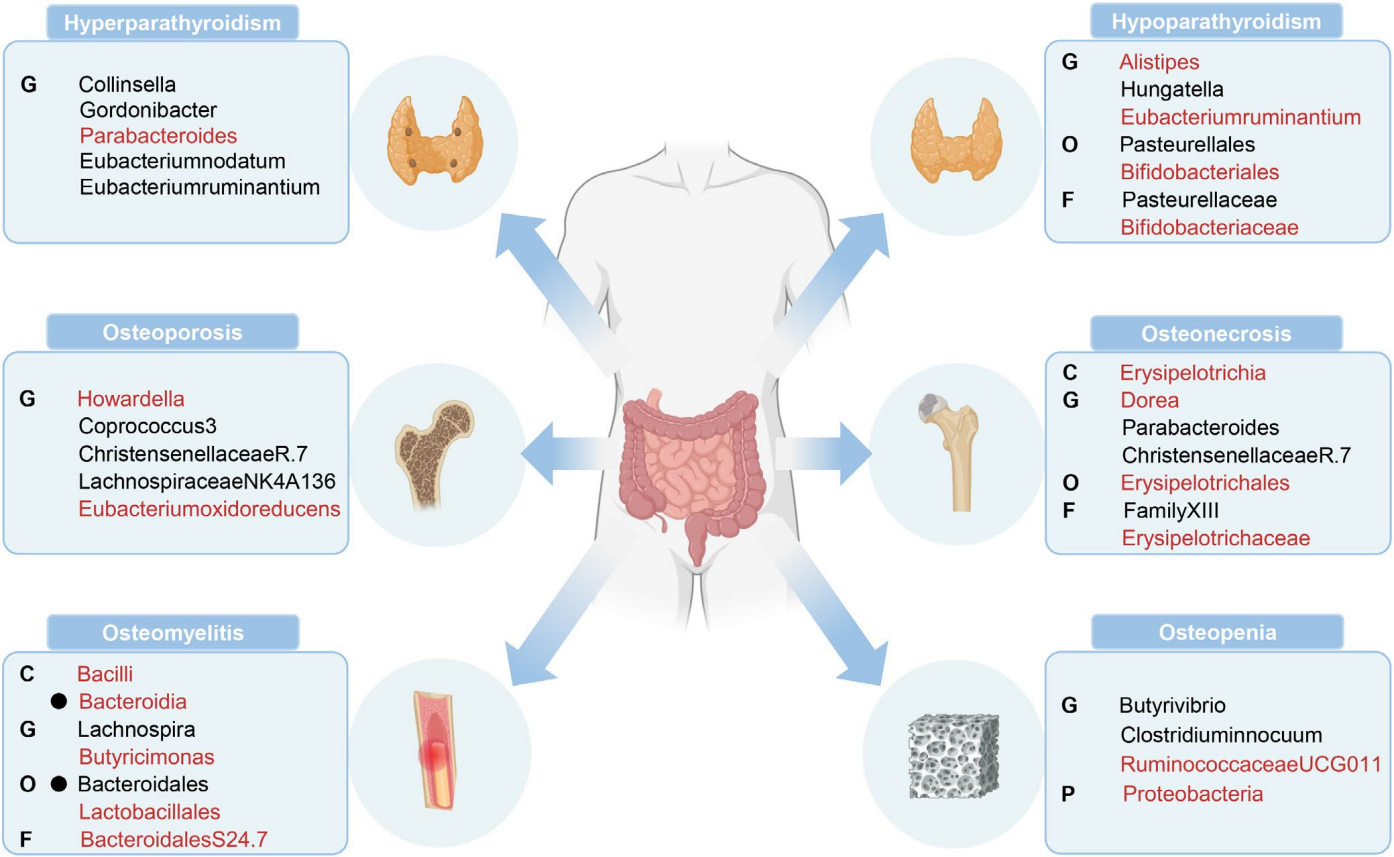
A schematic diagram of interrelationships of the relationship between gut microbiota and six metabolic bone diseases. Black colour indicates protective factors and red risk factors. Where solid dots indicate causality after reverse MR analysis. C, class; F, family; G, genus; O, order.

Moreover, reverse MR analysis suggested a potential increase in the abundance of class *Bacteroidia* and order *Bacteroidales* due to osteomyelitis (OR = 1.10, 95% CI = 1.02–1.19, *p*_FDR_ = 0.035). The Cochran's *Q*‐test of reverse MR analysis did not reveal significant heterogeneity, and both MR‐Egger and MR‐PRESSO analyses indicated no substantial levels of pleiotropy or outliers(*p* > 0.05), as documented in Table S6. A schematic diagram of interrelationships for a better understanding of the relationship between GM and six metabolic bone diseases is presented in Figure 5.

## 4 Discussion

In this bidirectional two‐sample MR study, our investigation centred on the complex causal relationships between the GM and six metabolic bone diseases. Our findings identified 35 significant causal relationships: three positives and two negatives with osteoporosis; two positives and two negatives with osteopenia; three positives and four negatives with osteonecrosis; two positives and five negatives with osteomyelitis; three positives and four negatives with hypoparathyroidism; and four positives and one negative with hyperparathyroidism. Co‐localisation analyses showed no pleiotropy or LD in the above causal relationships, which strongly support an aetiological link between the transgene and these bone diseases. Furthermore, reverse MR analysis revealed significant relationships between Bacteroidia (class) and Bacteroidales (order) with osteomyelitis, adjusted for FDR. These findings underscore a significant link between specific genetic alterations and metabolic bone diseases, reinforcing the gut–bone axis theory and offering new insights into how the GM influences the development of these conditions.

The GM is currently recognised as a pivotal element in the regulation of inflammation, involving different diseases [38] and diverse functions [39]. These include participation in the physiological functions of the gut itself, nutrient absorption and metabolism, host growth, metabolic regulation, immune system functionality, brain behaviour systems and cascade inflammatory processes [40]. In our MR analysis exploring the relationship between GM and osteomyelitis, the protective associations observed with the class *Bacilli* S24.7 and genus *Lachnospira* suggest potential roles in bolstering bone against infections. Conversely, increased risks were associated with class *Bacilli*, class *Bacteroidia*, genus *Butyricimonas* and specific orders. Although ubiquitous in the environment and mostly non‐invasive, Bacillus species like *Bacillus cereus* were found to produce toxins such as hemolysins, phospholipases and proteases, which are linked to the pathogenicity of osteomyelitis [41, 42]. Various studies highlight the regulating role of *Bacilli*, specifically *Bacillus cereus* , in osteomyelitis cases [43]. This aligns with our findings that an increased abundance of *Bacilli* is a potential risk factor for osteomyelitis. Additionally, we found a nominal significance in the reverse MR analysis between class *Bacteroidia* and order *Bacteroidales* with osteomyelitis, suggesting a bidirectional causality. Studies found *Bacteroidales* prevalent in bacterial osteomyelitis cases and rheumatoid arthritis‐related osteomyelitis [44], with recent microbiome analysis confirming their higher abundance in non‐healing diabetic foot ulcers [45]. This aligns with our reverse MR analysis findings and elucidates the intricate relationship between GM and osteomyelitis within the gut–bone axis framework. The state of the GM influences the host's resistance to osteomyelitis, suggesting that altering the microbiome could be an effective method to mitigate osteomyelitis risk, thus meriting further investigation into the underlying mechanisms [46].

It is known that intestinal calcium absorption is crucial for maintaining bone mineralisation [47], and our study also revealed some significant causal relationships between GM and metabolic bone diseases such as osteoporosis and osteopenia. Genera such as *Coprococcus3* and *Lachnospiraceae NK4A136* were found to be protective in the maintenance of bone density, in contrast to *Howardella* and *Ruminococcaceae UCG011*, which were associated with a higher risk, and these associations deepen our understanding of the gut–skeletal axis. The GM potentially impacts calcium absorption, osteoclast and osteoblast activity and overall bone turnover, particularly affecting calcium –phosphorus metabolism [48]. This influence extends to the regulation of crucial metabolic pathways involved in bone health, such as those governed by calcium and phosphorus cycles.

The calcium and phosphorus cycles, pivotal for bone health and integral to the gut–bone axis, are significantly influenced by the GM. This impact is particularly pronounced in the biosynthesis and bioavailability of vitamins and hormones, such as vitamin D, which is essential for calcium and phosphorus metabolism. This metabolic pathway is closely interconnected with the function of parathyroid hormone (PTH), a crucial single‐chain peptide hormone. PTH directly influences bones and kidneys, regulating the renal reabsorption of calcium and phosphorus, and playing a key role in mobilising calcium from bones into the bloodstream. The interaction between GM and PTH is evident in the regulation of bone formation and mass. For instance, germ‐free mice lacking microbiota have demonstrated that reduced butyrate levels lead to impaired PTH function in bone regulation, while the reintroduction of butyrate restores this crucial function, highlighting the vital role of GM in effectively regulating PTH and, by extension, calcium –phosphorus metabolism [49]. In this context, our study found that the *Bifidobacteriaceae* are a risk factor for hyperparathyroidism, while *Pasteurellales* and *Eubacterium ruminantium* demonstrate contrasting associations with parathyroid disorders, acting as both protective and risk factors. These findings suggest a complex interplay between GM dysbiosis, calcium‐phosphorus metabolism and PTH function, contributing to our understanding of their combined influence on bone‐related diseases.

Further highlighting the multifaceted mechanisms of GM in bone health regulation, microbial metabolic products, particularly short‐chain fatty acids (SCFAs) like butyrate, propionate and acetate, play a significant role [50]. These SCFAs, primarily produced from the metabolism of indigestible dietary fibres in food, increase the solubility and absorption of calcium by reducing the intestinal pH and hindering the formation of intestinal calcium‐phosphate complexes [51]. Studies have shown that propionate and butyrate inhibit osteoclast differentiation and bone resorption by downregulating TRAF6 and NFATc1 [52]. Additionally, butyrate directly induces metabolic reprogramming in osteoclast precursors, enhancing glycolysis and downregulating key osteoclast genes, thereby preventing osteoclast differentiation [53]. SCFAs also promote the differentiation of CD4+ T cells into Treg cells, reducing AP‐1 and facilitating NFAT and SMAD signalling transcription, activating Wnt10b and thus stimulating osteoblast Wnt signalling [54]. The promotion of bone synthesis by PTH also requires the action of butyrate, suggesting its potential regulatory effect on calcium‐phosphate levels and bone metabolism [49]. Overall, SCFAs are vital regulators in maintaining bone homeostasis. Moreover, treating obese/T2D mice with inulin‐rich chicory fructans significantly reduced the severity of acute prosthetic joint infections caused by *Staphylococcus aureus* , indicating that microbial metabolites can control distant bacterial infections and may play a supplementary role in the treatment of osteomyelitis [55].

As exploration into the relationship between GM and host bone metabolism continues, GM has also been shown to potentially modulate bone metabolism by influencing local and systemic immune systems [56]. Regulatory T cells (Tregs) inhibit osteoclast differentiation and bone resorption by reducing IL‐17 expression, whereas Th17 cells promote osteoclast differentiation by activating the RANKL pathway, which together affect bone metabolism and disrupt bone immunity, which is critical for maintaining bone homeostasis [57]. Previous studies have shown that reduced *Lactobacillus animalis* abundance was associated with the development of GC‐induced ONFH in mice, and that oral administration of *L. animalis* at an early stage of GC exposure effectively protects the femoral head from GC‐induced osteonecrosis [58]. It is thus clear that enriching GM can regulate microbial metabolic products, immune modulation, intestinal mucosal barrier and endocrine regulation, thereby modulating bone metabolism [59]. Our study found that genera such as *Christensenellaceae R.7* and *Parabacteroides* are protective against a variety of metabolic bone diseases such as osteoporosis and osteonecrosis, while *Erysipelotrichia* and *Erysipelotrichia* are associated with an increased risk of osteonecrosis. Notably, overgrowth of certain microbes or a substantial reduction in others can lead to disruption of the gut microbial ecosystem, loss of key physiological functions and a significant impact on intestinal calcium‐phosphorus absorption and bone metabolism [60].

This study highlights the critical role of GM in influencing bone health through the gut–bone axis, indicating the potential to develop targeted interventions for metabolic bone diseases by modulating GM. Utilising extensive data, our research analysed the causal effects of various taxonomic groups on bone diseases, effectively minimising confounding factors in epidemiology. Our approach, incorporating stringent quality control and sensitivity analyses, enhanced the credibility of our MR study. In addition, we applied co‐localisation analysis to provide a valuable basis for MR analysis to further validate possible confounders and pleiotropy issues and to strengthen the results of MR analysis.

However, our study also has limitations. It predominantly analysed individuals of European descent, necessitating further research to ascertain if similar genetic variations exist in other ethnic groups. Another limitation includes the relatively small size of some exposed GWAS, though the statistical power of MR largely depends on the strength of SNP‐result associations. We adopted a *p*‐value threshold of < 10^−5^ in selecting instrumental variables. While this might increase the risk of weak instrument bias for individual genetic variants, our F‐statistics indicate these instruments have adequate strength (all above 10), suggesting that lowering the *p*‐value threshold should enhance the overall statistical power of MR analyses. Therefore, replicating our analyses with larger‐scale GWAS, including those with more detailed disease phenotypic data, would be beneficial. Additionally, the lack of detailed phenotypic data in existing GWAS precluded examining associations with specific types of cases, such as initial onset or treatment tolerance. Future larger‐scale global genome studies may help investigate these potential associations.

Emphasising the reduction of biases in observational studies, future research should focus on deciphering the mechanisms behind these associations for clinical application.

## 5 Conclusion

Our MR analysis investigates the causal relationships between 211 GM and six metabolic bone diseases. This study reveals many significant causal associations between genetic modifications and a range of metabolic bone diseases, highlighting the critical role of genetic modifications in bone metabolism and bone disease. These significant findings pave the way for further exploration into the complex biological interactions between GM and bone health, and they provide direction for future therapeutic interventions targeting the gut–bone axis.

## Author Contributions

**Yanling Zhou:** methodology (equal), writing – original draft (equal). **Yao Yang:** methodology (equal), writing – original draft (equal). **Wanbo Zhu:** conceptualization (equal). **Nikolaos Kourkoumelis:** data curation (equal). **Yingjie Wang:** resources (equal). **Yuan Chen:** data curation (lead). **Lingxiang Hong:** resources (equal). **Junjie Wang:** investigation (lead). **Junchen Zhu:** writing – review and editing (equal). **Chen Zhu:** writing – review and editing (equal). **Xianzuo Zhang:** writing – review and editing (lead).

## Ethics Statement

The datasets used in the current study were publicly available, and ethical approval and informed consent were obtained prior to implementation. Therefore, our study did not require any additional informed consent or ethical approval.

## Conflicts of Interest

The authors declare no conflicts of interest.

## Supporting information


Data S1.

Data S2.

## Data Availability

The datasets analysed in the current study can be downloaded from the website https://mibiogen.gcc.rug.nl/, https://www.nealelab.is/uk‐biobank, https://www.finngen.fi/en/access_results.
