# Supplementary material for: Microbial Influences on Calcium‐Phosphorus Homeostasis and Metabolic Bone Diseases: A Bidirectional Mendelian Randomisation Study on the Gut–Bone Axis
Source: J Cell Mol Med. 2025 Apr 1;29(7):e70491. doi: 10.1111/jcmm.70491 (PMC11959414; doi:10.1111/jcmm.70491)
Supplement: Supplementary file 1 — Data S1. [file JCMM-29-e70491-s002.pdf]

Supplementary Information

Microbial Influences on Calcium-Phosphorus Homeostasis and Metabolic Bone Diseases: A Bidirectional Mendelian Randomization Study on the Gut-Bone Axis

Yanling Zhou[a b1], *Yao Yang*[b1], *Wanbo Zhu*[c], *Yingjie Wang*[b], *Yuan Chen*[b], *Lingxiang Hong*[b], *Junjie Wang*[b], *Junchen Zhu*[b *], *Chen Zhu*[a *], *Xianzuo Zhang*[a *]

[a] Department of Orthopedics, The First Affiliated Hospital of USTC, Division of Life Sciences and Medicine, University of Science and Technology of China, Hefei, Anhui, 230001, China.

[b] Department of Orthopedics, The Second Affiliated Hospital of Anhui University of Chinese Medicine, Hefei 230001, China.

[c] Department of Orthopedics, Shanghai Sixth People's Hospital Affiliated to Shanghai,

Jiao Tong University School of Medicine, Shanghai Jiao Tong University, Sha

[1] These authors contribute equally to this manuscript.

* Correspondence and requests for materials should be addressed to

Xianzuo Zhang
Department of Orthopedics, Centre for Leading Medicine and Advanced Technologies of IHM, The First Affiliated Hospital of USTC, Division of Life Sciences and Medicine, University of Science and Technology of China, Hefei 230022, China. **E-mail: zhangxianzuo@foxmail.com**

Chen Zhu
Department of Orthopedics, Centre for Leading Medicine and Advanced Technologies of IHM, The First Affiliated Hospital of USTC, Division of Life Sciences and Medicine, University of Science and Technology of China, Hefei 230022, China. **E-mail: zhuchena@ustc.edu.cn**

Junchen Zhu
Department of Orthopedics, The Second Affiliated Hospital of Anhui University of Chinese Medicine, Hefei 230001, China. **E-mail: 2006zhujc@163.com**

Figures S1. Leave-one-out plots of significant and nominally significant estimates of genetically predicted gut microbiota on osteoporosis;

Figures S2. Leave-one-out plots of significant and nominally significant estimates of genetically predicted gut microbiota on osteopenia;

Figures S3. Leave-one-out plots of significant and nominally significant estimates of genetically predicted gut microbiota on osteonecrosis;

Figures S4. Leave-one-out plots of significant and nominally significant estimates of genetically predicted gut microbiota on osteomyelitis;

Figures S5. Leave-one-out plots of significant and nominally significant estimates of genetically predicted gut microbiota on hypoparathyroidism;

Figures S6. Leave-one-out plots of significant and nominally significant estimates of genetically predicted gut microbiota on hyperparathyroidism;

Figures S7. Scatterplot of genetically predicted gut microbiota on osteoporosis;

Figures S8. Scatterplot of genetically predicted gut microbiota on osteopenia;

Figures S9. Scatterplot of genetically predicted gut microbiota on osteonecrosis;

Figures S10. Scatterplot of genetically predicted gut microbiota on osteomyelitis;

Figures S11. Scatterplot of genetically predicted gut microbiota on hypoparathyroidism;

Figures S12. Scatterplot of genetically predicted gut microbiota on hyperparathyroidism;

Figures S13. LocusZoom and LocusCompare plots of significant and nominal significant estimates from genetically predicted gut microbiota on six metabolic bone diseases.

Supplement Figure S1

Leave-one-out plots of significant and nominal significant estimates from genetically predicted gut microbiota {(A)genus..Eubacteriumoxidoreducens;(B)genus.ChristensenellaceaeR.7;(C)genus.Coprococcus3;(D)genus.Howardella;(E) genus.LachnospiraceaeNK4A136} on Osteoporosis

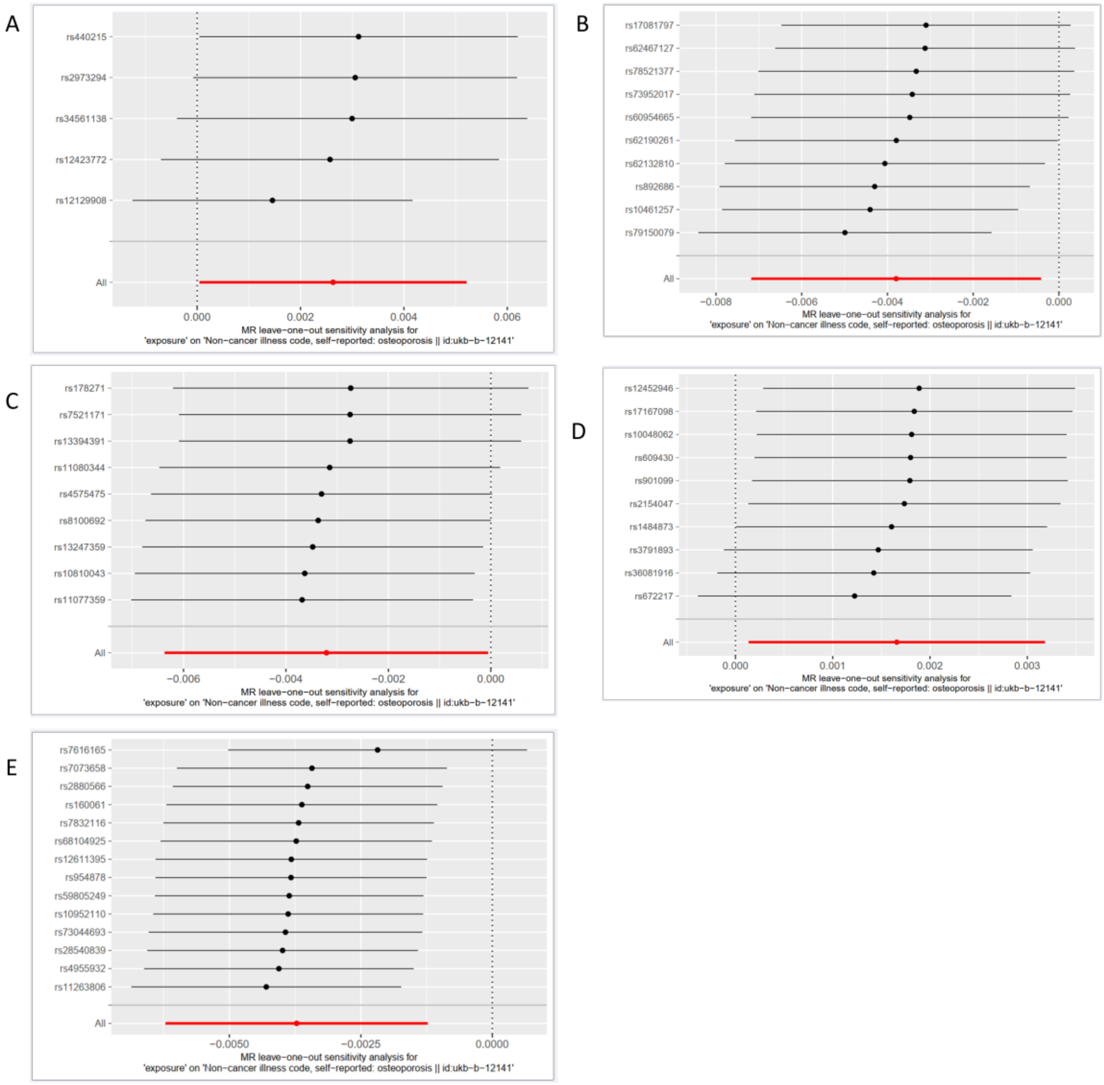

Supplement Figure S2

Leave-one-out plots of significant and nominal significant estimates from genetically predicted gut microbiota {genus..Clostridiuminnocuum;(B)genus.Butyrivibrio;(C)genus.RuminococcaceaeUCG011;(D)phylum.Proteobacteria} on Osteopenia

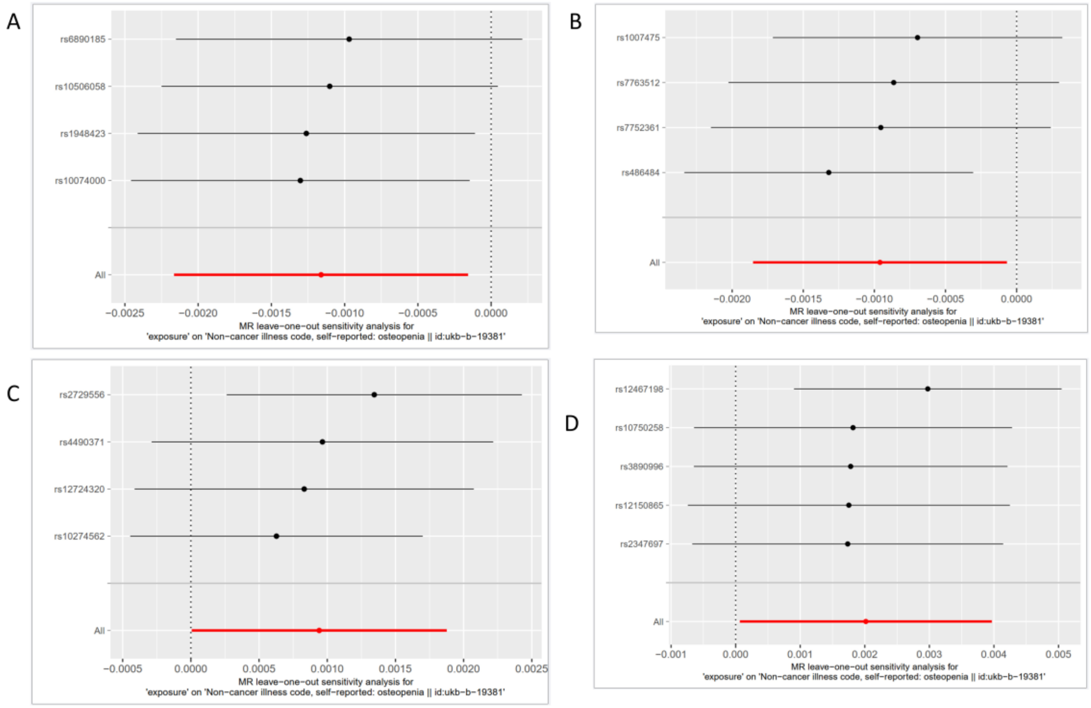

Supplement Figure S3

Leave-one-out plots of significant and nominal significant estimates from genetically predicted gut microbiota{(A)class.Erysipelotrichia;(B)family.Erysipelotrichaceae;(C)family.Family XIII;(D)genus.ChristensenellaceaeR.7;(E)genus.Dorea;(F)genus.Parabacteroides;(G)order.Erysipelotrichales} on Osteonecrosis

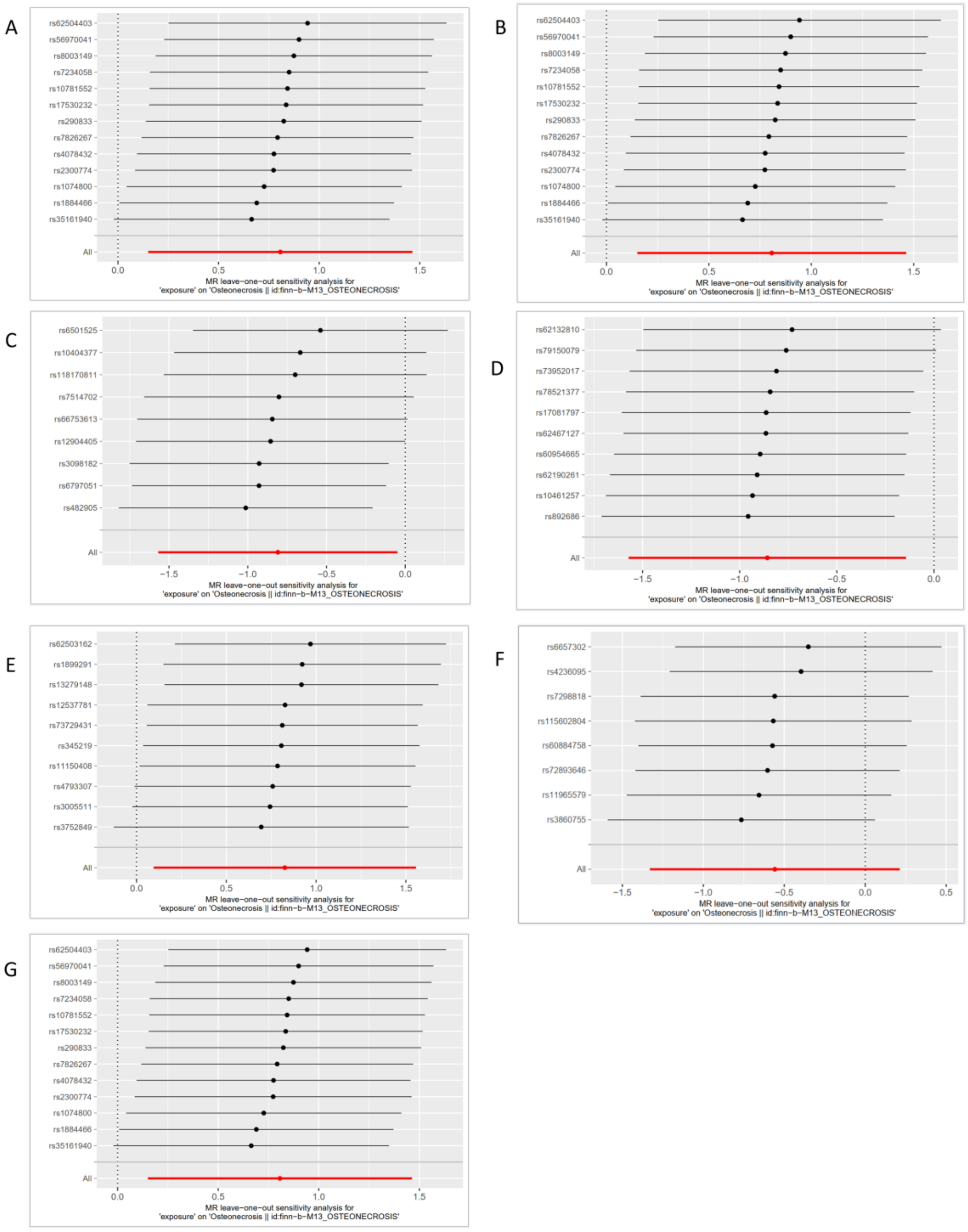

Supplement Figure S4

Leave-one-out plots of significant and nominal significant estimates from genetically predicted gut microbiota {(A)class.Bacilli;(B)class.Bacteroidia;(C)family.BacteroidalesS24.7;(D)genus.Butyricimonas;(E)genus.Lachnospira;(F)order.Bacteroidales; (G) order.Lactobacillales} on Osteomyelitis

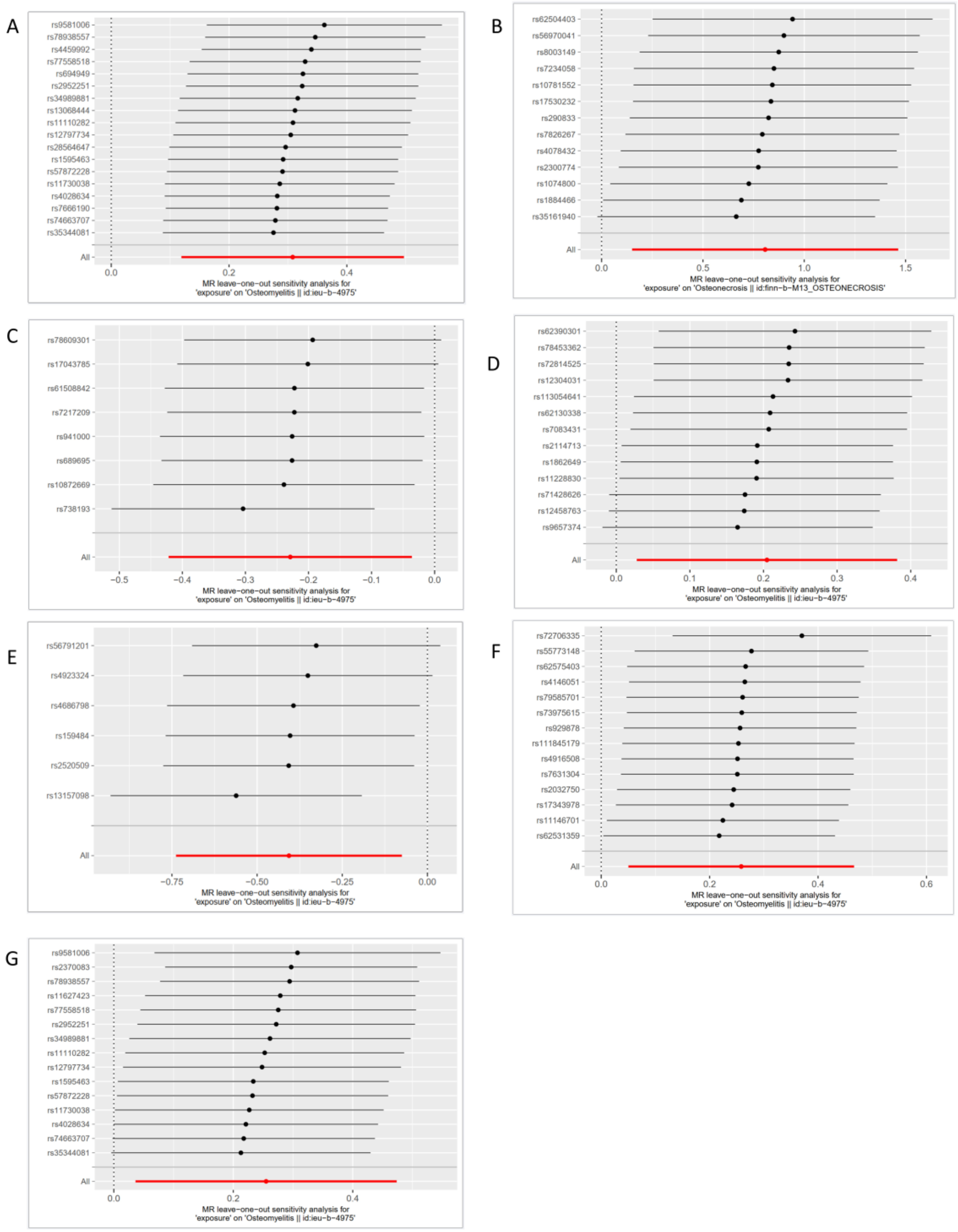

Supplement Figure S5

Leave-one-out plots of significant and nominal significant estimates from genetically predicted gut microbiota {(A) family.Bifidobacteriaceae;(B)family.Pasteurellaceae;(C)genus..Eubacteriumruminantium;(D)genus.Alistipes;(E)genus.Hungatella;(F)order.Bifidobacteriales;(G)order.Pasteurellales} on Hypoparathyroidism

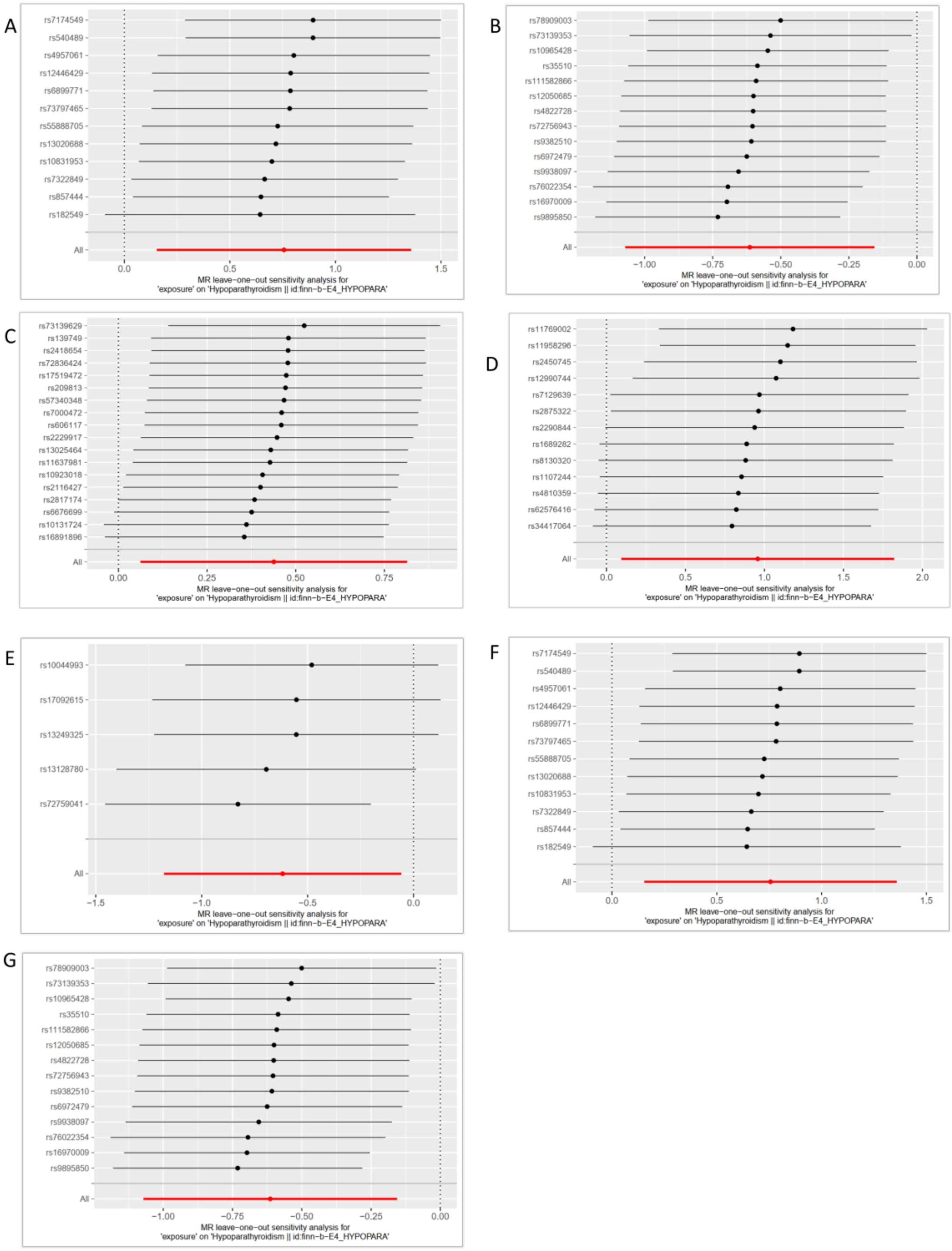

Supplement Figure S6

Leave-one-out plots of significant and nominal significant estimates from genetically predicted gut microbiota {(A)genus..Eubacteriumnodatum;(B)genus..Eubacteriumruminantium;(C)genus.Collinsella;(D)genus.Gordonibacter;(E)genus.Parabacteroides} on Hyperparathyroidism

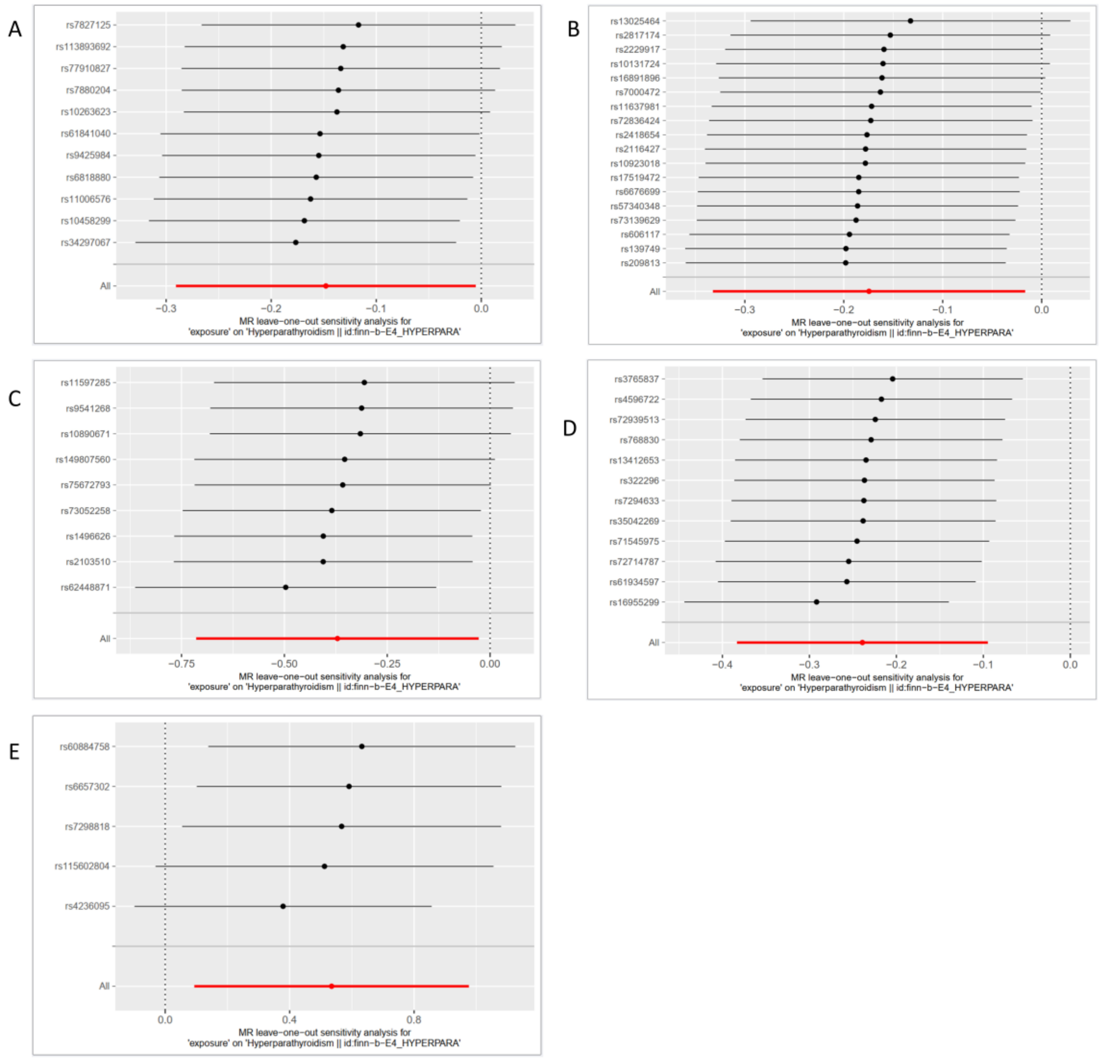

Supplement Figure S7

Scatter plots of significant and nominal significant estimates from genetically predicted gut microbiota{(A)genus..Eubacteriumoxidoreducens;(B)genus.ChristensenellaceaeR.7;(C)genus.Coprococcus3;(D)genus.Howardella; (E) genus.LachnospiraceaeNK4A136} on Osteoporosis

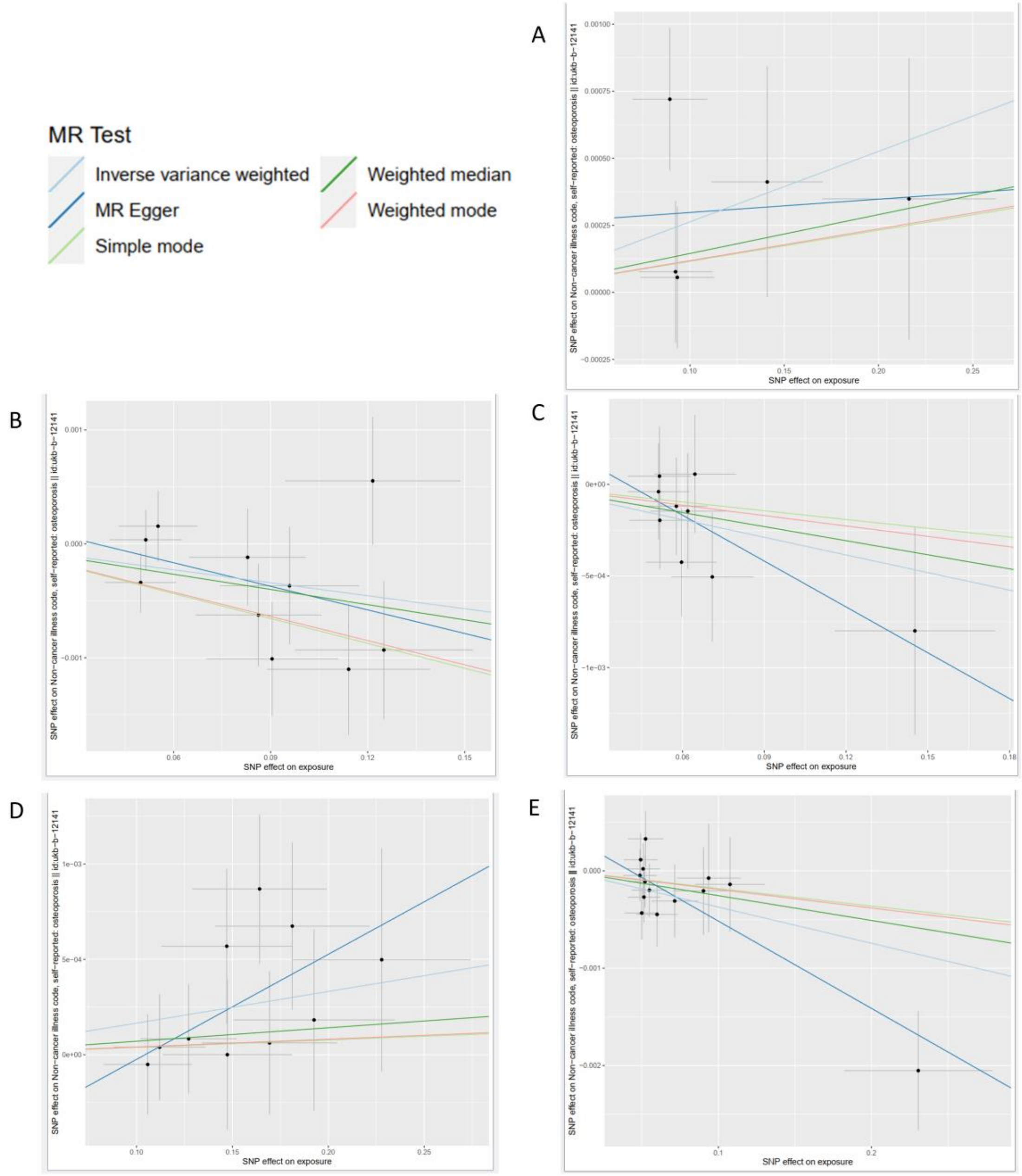

Supplement Figure S8

Scatter plots of significant and nominal significant estimates from genetically predicted gut microbiota{(A)genus..Clostridiuminnocuum;(B)genus.Butyrivibrio;(C)genus.Ruminococcaceae UCG011;(D)phylum.Proteobacteria} on Osteopenia

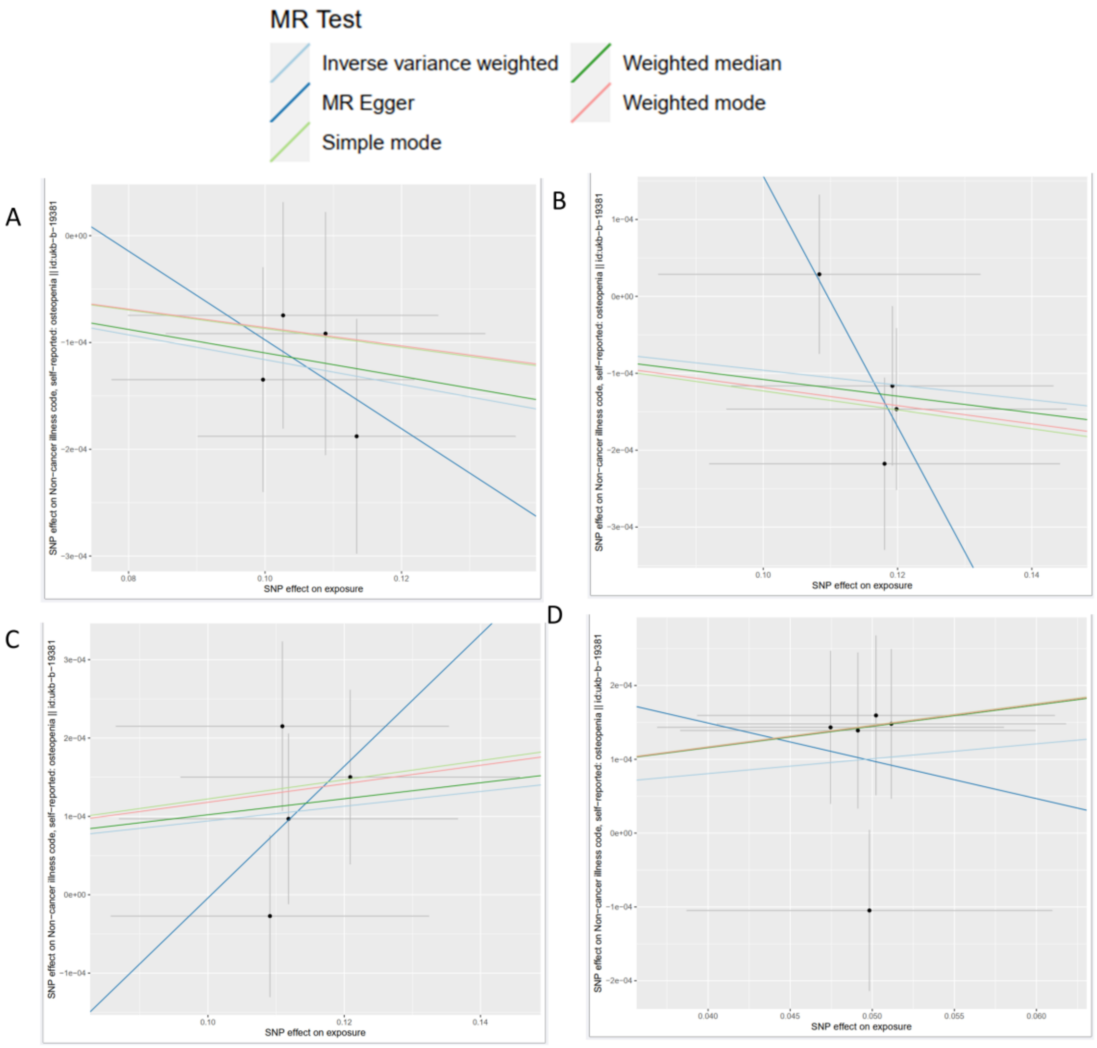

Supplement Figure S9

Scatter plots of significant and nominal significant estimates from genetically predicted gut microbiota{(A)class.Erysipelotrichia;(B)family.Erysipelotrichaceae;(C)family.Family XIII;(D)genus.ChristensenellaceaeR.7;(E)genus.Dorea;(F)genus.Parabacteroides;(G)order.Erysipelotrichales} on Osteonecrosis

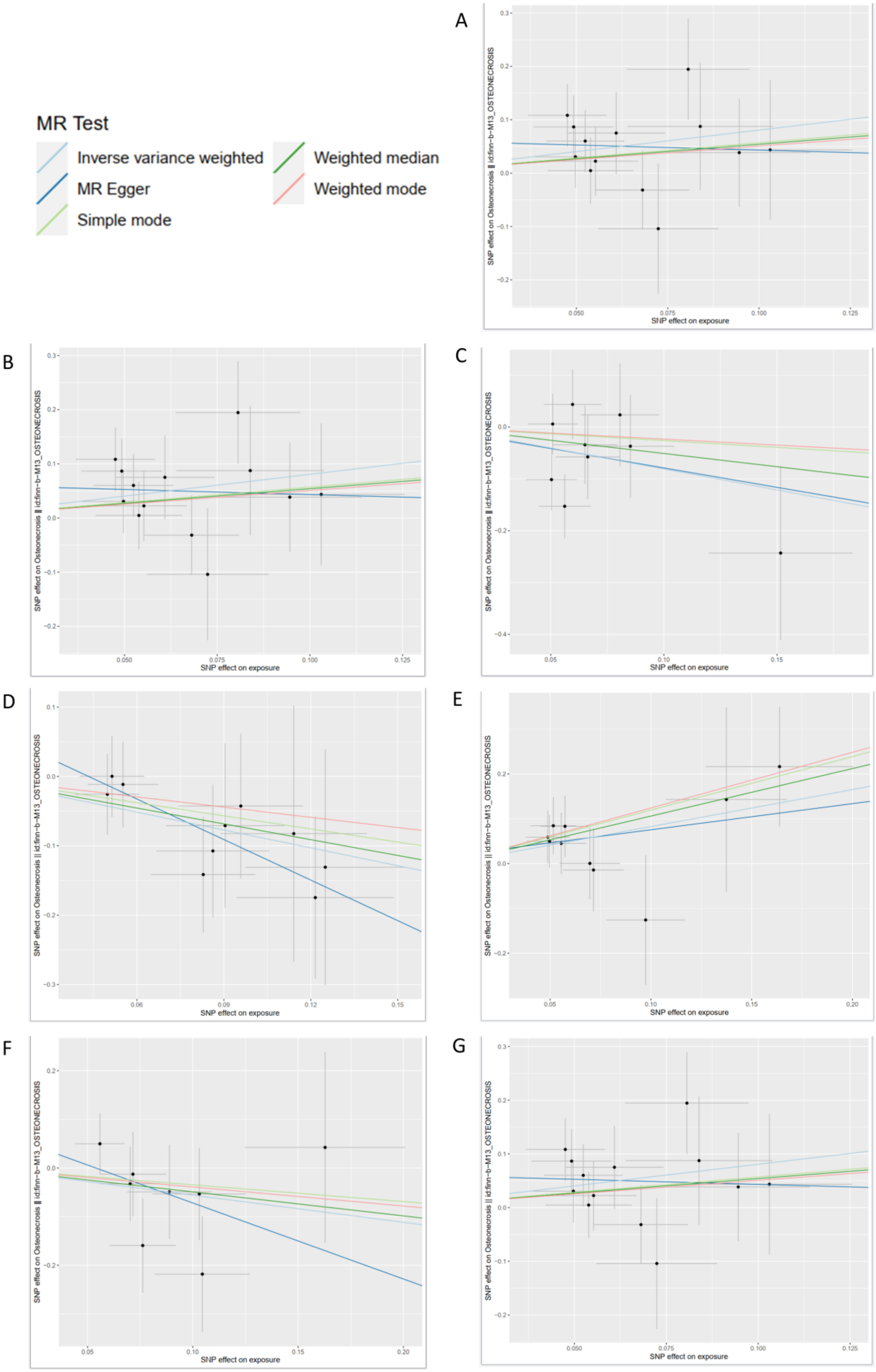
MR Test
Inverse variance weighted
MR Egger
Simple mode
Weighted median
Weighted mode
A
B
C
D
E
F
G
SNP effect on exposure
SNP effect on Osteonecrosis || id:finn-b-M13_OSTEONECROSIS

Supplement Figure S10

Scatter plots of significant and nominal significant estimates from genetically predicted gut microbiota{(A)class.Bacilli;(B)class.Bacteroidia;(C)family.BacteroidalesS24.7;(D)genus.Butyricimonas;(E)genus.Lachnospira;(F)order.Bacteroidales; (G) order.Lactobacillales} on Osteomyelitis

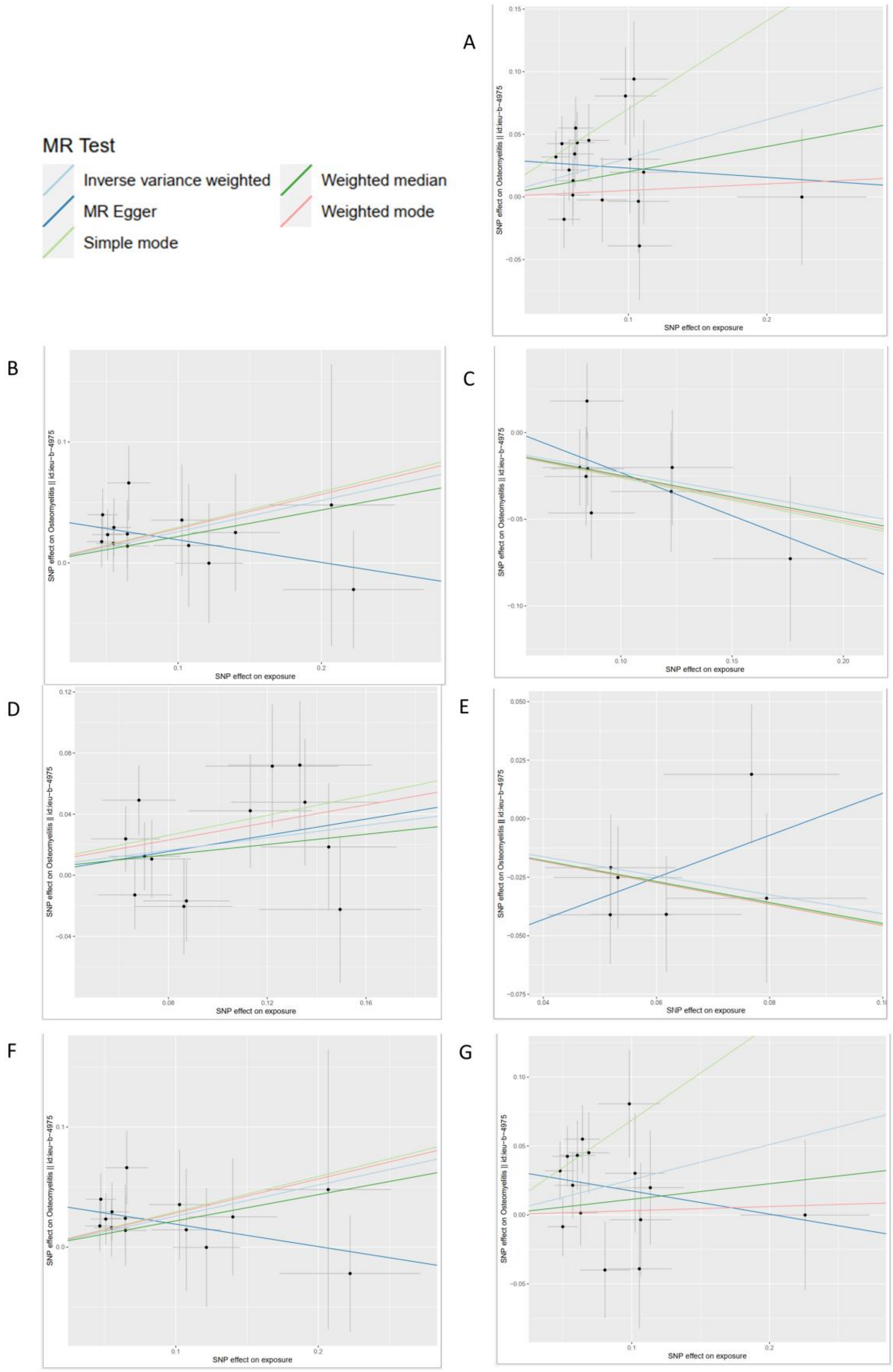
MR Test
Inverse variance weighted
MR Egger
Simple mode
Weighted median
Weighted mode
A
B
C
D
E
F
G
SNP effect on Osteomyelitis || id:ieu-b-4975
SNP effect on exposure

Supplement Figure S11

Scatter plots of significant and nominal significant estimates from genetically predicted gut microbiota{(A)family.Bifidobacteriaceae;(B)family.Pasteurellaceae;(C)genus..Eubacteriumruminantium;(D)genus.Alistipes;(E)genus.Hungatella;(F)order.Bifidobacteriales;(G)order.Pasteurellales} on Hypoparathyroidism

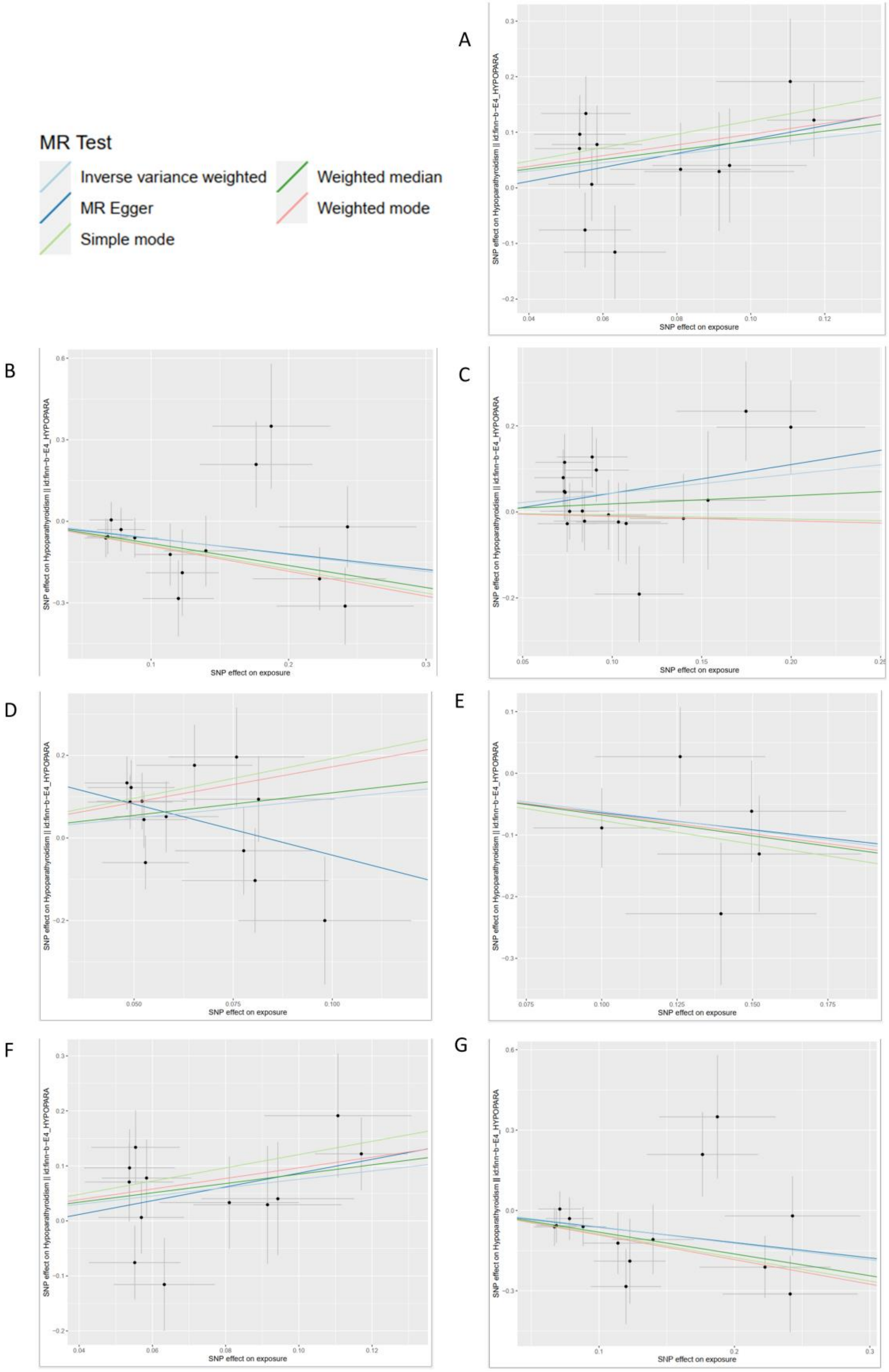

MR Test
Inverse variance weighted
MR Egger
Simple mode
Weighted median
Weighted mode
A
B
C
D
E
F
G
SNP effect on Hypoparathyroidism || id:finn-b-E4_HYPOPARA
SNP effect on exposure

Supplement Figure S12

Scatter plots of significant and nominal significant estimates from genetically predicted gut microbiota{(A)genus..Eubacteriumnodatum;(B)genus..Eubacteriumruminantium;(C)genus.Collinsella;(D)genus.Gordonibacter;(E)genus.Parabacteroides} on Hyperparathyroidism

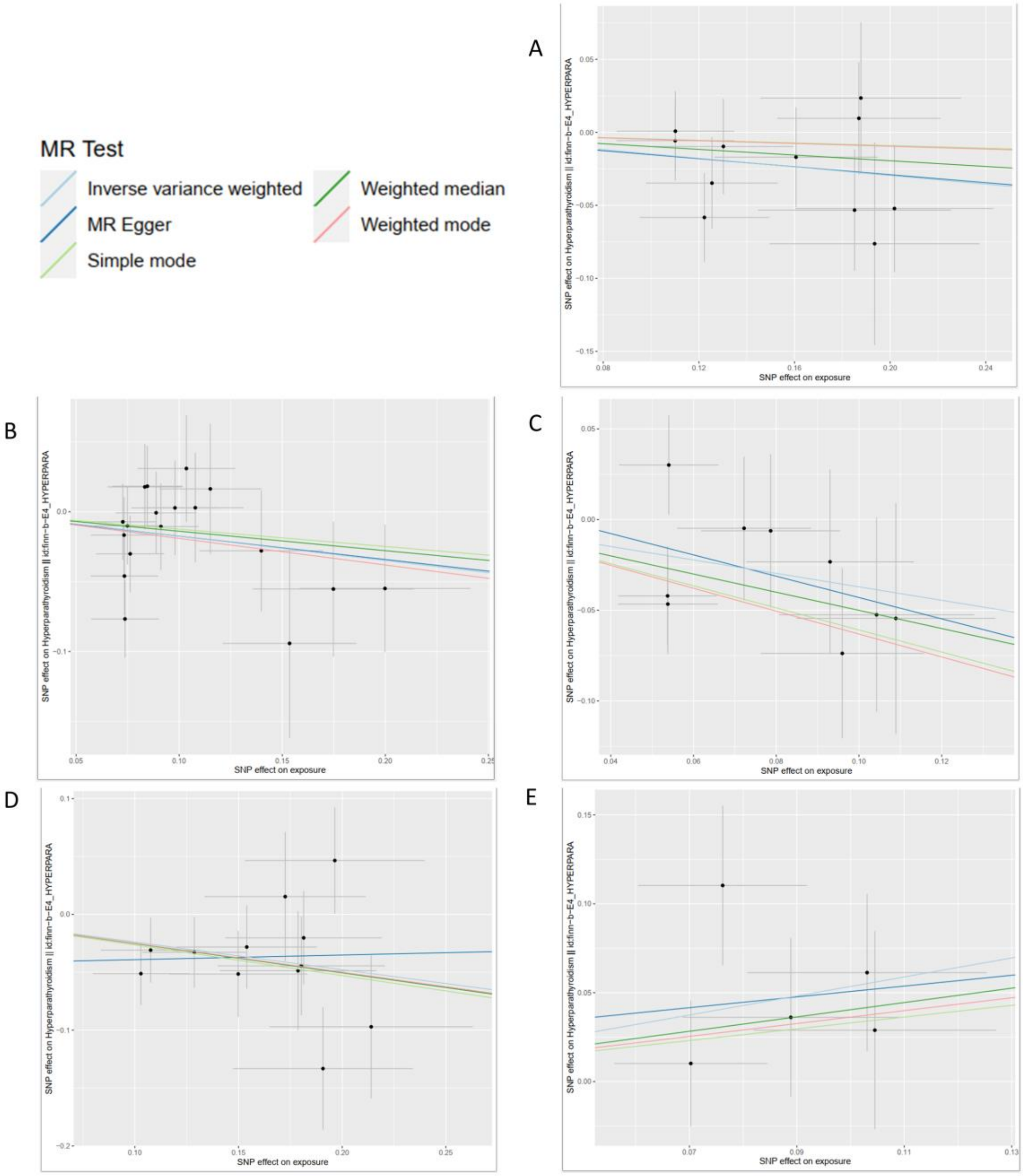

Supplement Figure S13

LocusZoom and LocusCompare plots of significant and nominal significant estimates from genetically predicted gut microbiota on six metabolic bone diseases.

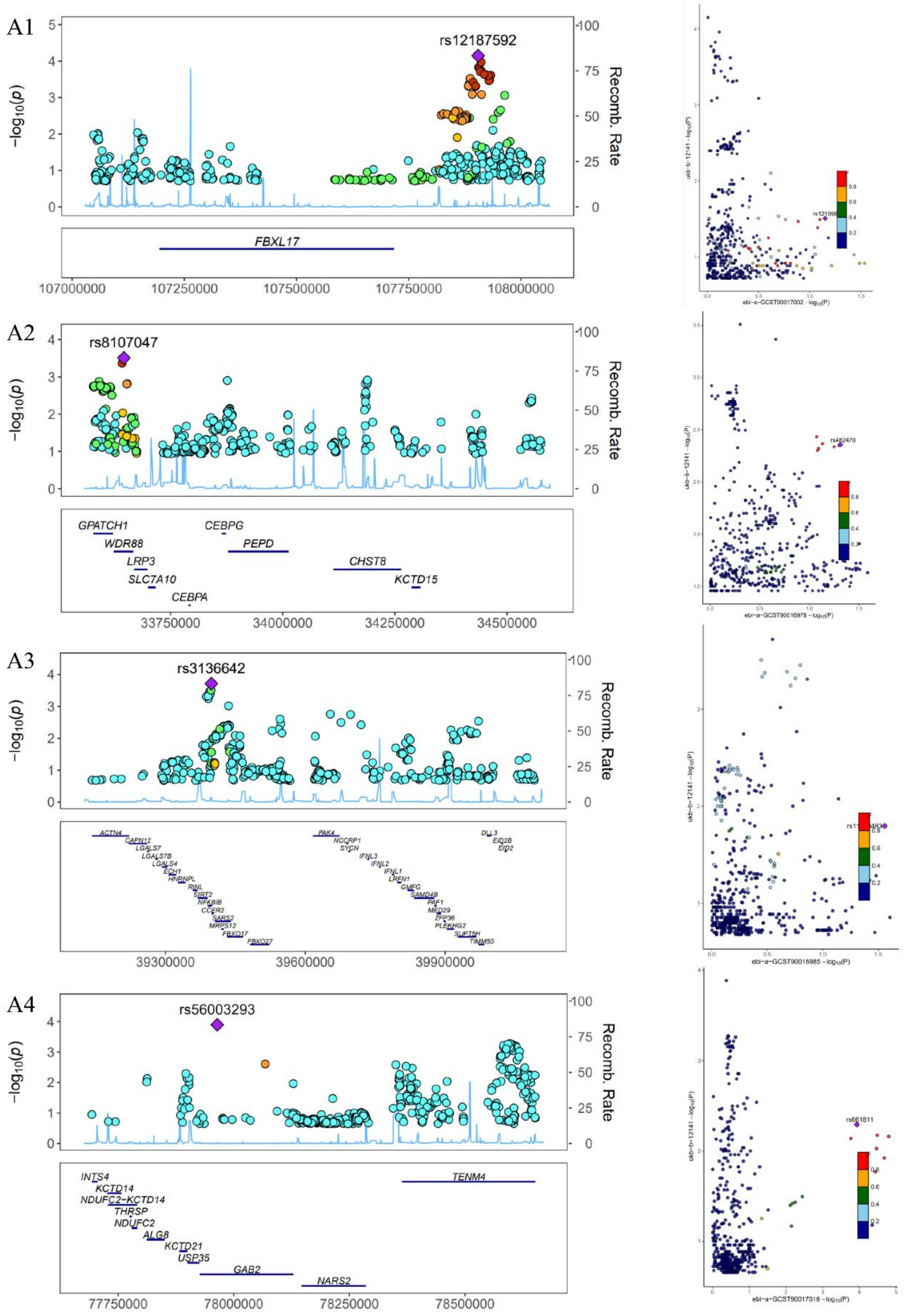

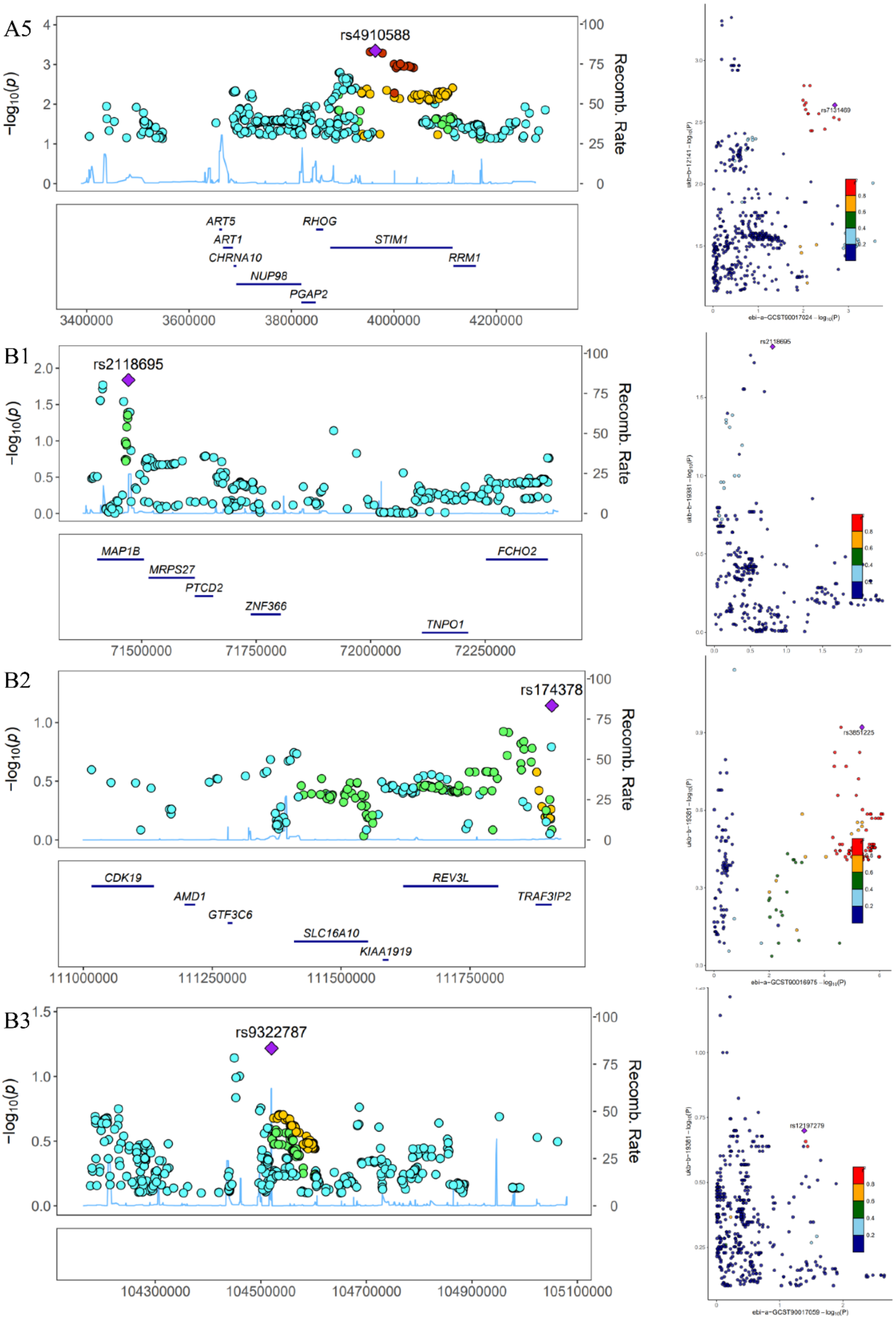

A5
rs4910588
−log10(p)
Recomb. Rate
ART5
RHOG
ART1
STIM1
CHRNA10
RRM1
NUP98
PGAP2
3400000
3600000
3800000
4000000
4200000
B1
rs2118695
MAP1B
FCHO2
MRPS27
PTCD2
ZNF366
TNPO1
71500000
71750000
72000000
72250000
B2
rs174378
CDK19
REV3L
AMD1
TRAF3IP2
GTF3C6
SLC16A10
KIAA1919
111000000
111250000
111500000
111750000
B3
rs9322787
104300000
104500000
104700000
104900000
105100000

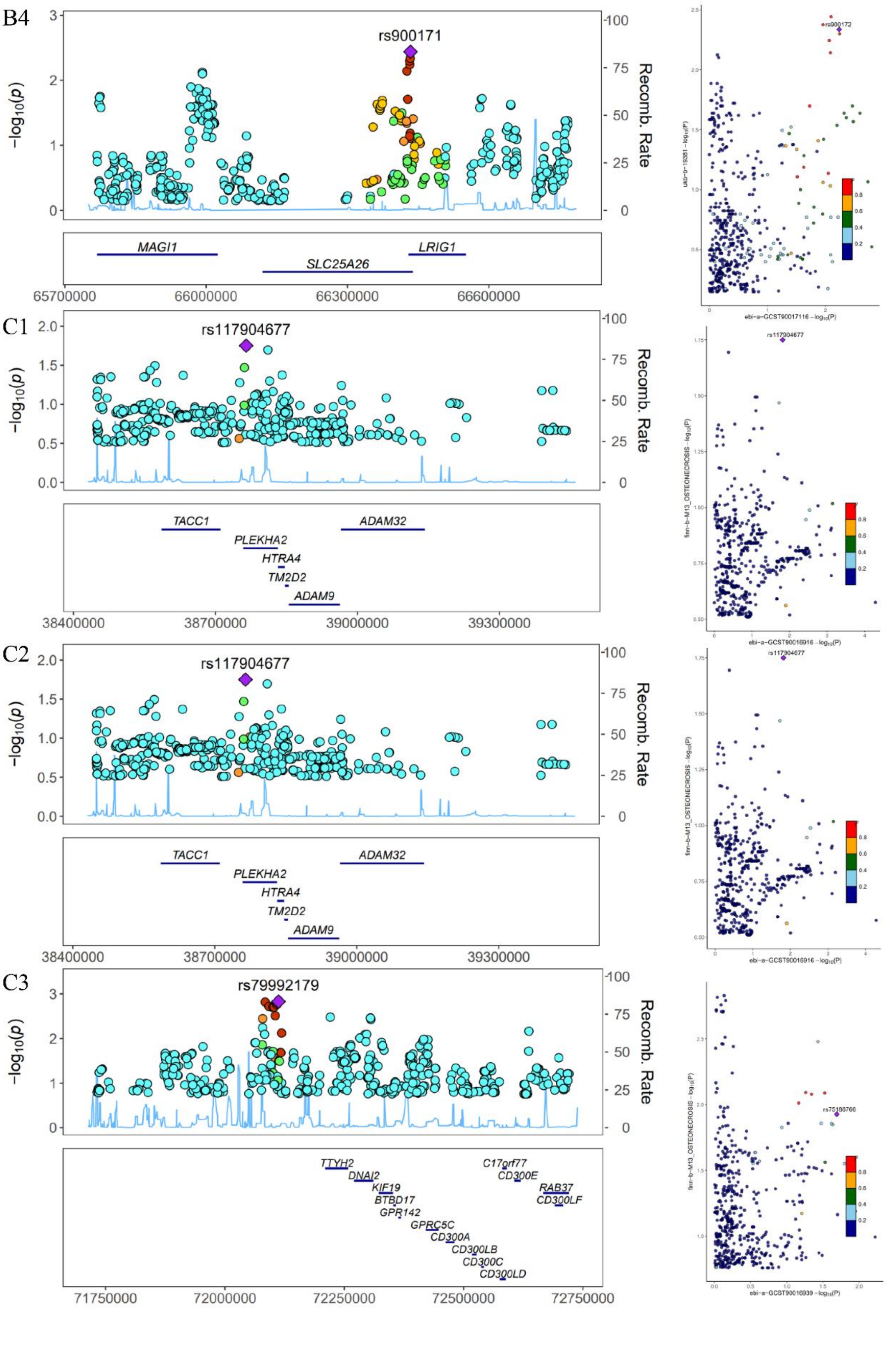

B4
rs900171
−log10(p)
Recomb. Rate
MAGI1
LRIG1
SLC25A26
65700000
66000000
66300000
66600000
rs900172
C1
rs117904677
TACC1
ADAM32
PLEKHA2
HTRA4
TM2D2
ADAM9
38400000
38700000
39000000
39300000
C2
rs117904677
TACC1
ADAM32
PLEKHA2
HTRA4
TM2D2
ADAM9
38400000
38700000
39000000
39300000
C3
rs79992179
TTYH2
DNAI2
KIF19
BTBD17
GPR142
GPRC5C
CD300A
CD300LB
CD300C
CD300LD
C17orf77
CD300E
RAB37
CD300LF
71750000
72000000
72250000
72500000
72750000
rs75186766

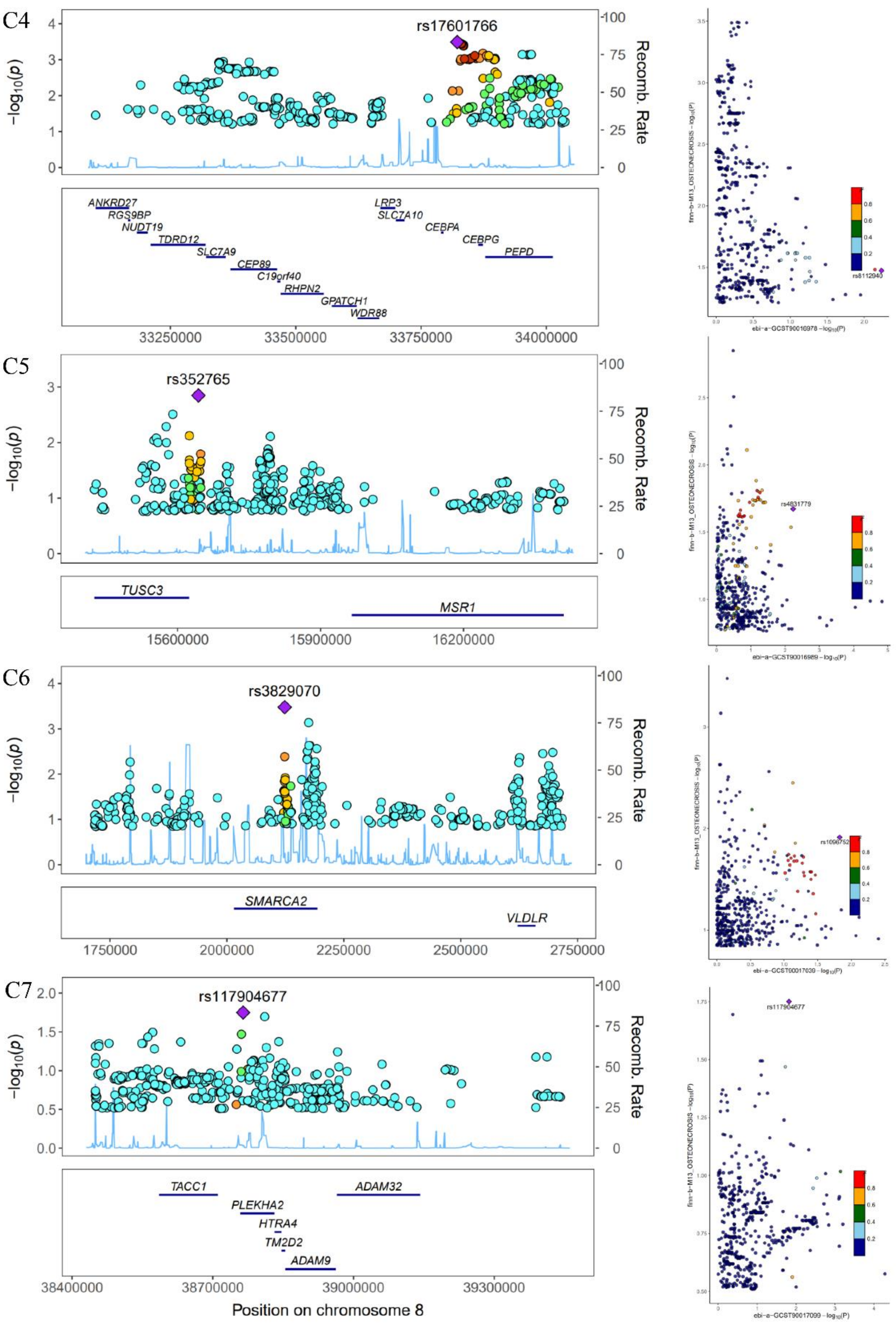
C4
rs17601766
−log10(p)
Recomb. Rate
ANKRD27
RGS9BP
NUDT19
TDRD12
SLC7A9
CEP89
C19orf40
RHPN2
GPATCH1
WDR88
LRP3
SLC7A10
CEBPA
CEBPG
PEPD
33250000
33500000
33750000
34000000
rs8112940
C5
rs352765
TUSC3
MSR1
15600000
15900000
16200000
rs4831779
C6
rs3829070
SMARCA2
VLDLR
1750000
2000000
2250000
2500000
2750000
C7
rs117904677
TACC1
PLEKHA2
HTRA4
TM2D2
ADAM9
ADAM32
38400000
38700000
39000000
39300000
Position on chromosome 8

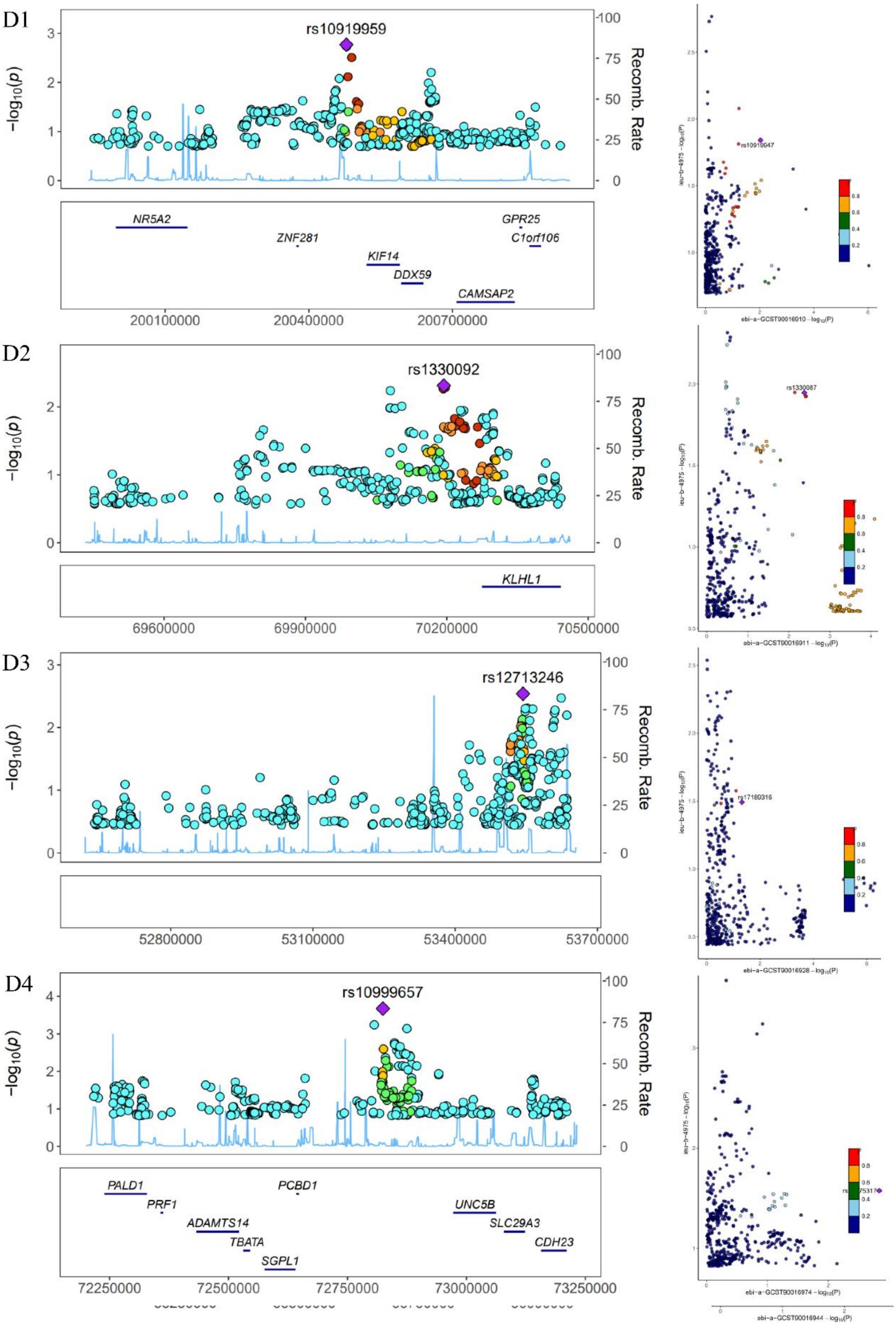

D1
rs10919959
-log10(p)
Recomb. Rate
NR5A2
ZNF281
KIF14
DDX59
CAMSAP2
GPR25
C1orf106
200100000
200400000
200700000
rs10919947
D2
rs1330092
KLHL1
69600000
69900000
70200000
70500000
rs1330087
D3
rs12713246
52800000
53100000
53400000
53700000
rs17180316
D4
rs10999657
PALD1
PRF1
ADAMTS14
TBATA
SGPL1
PCBD1
UNC5B
SLC29A3
CDH23
72250000
72500000
72750000
73000000
73250000

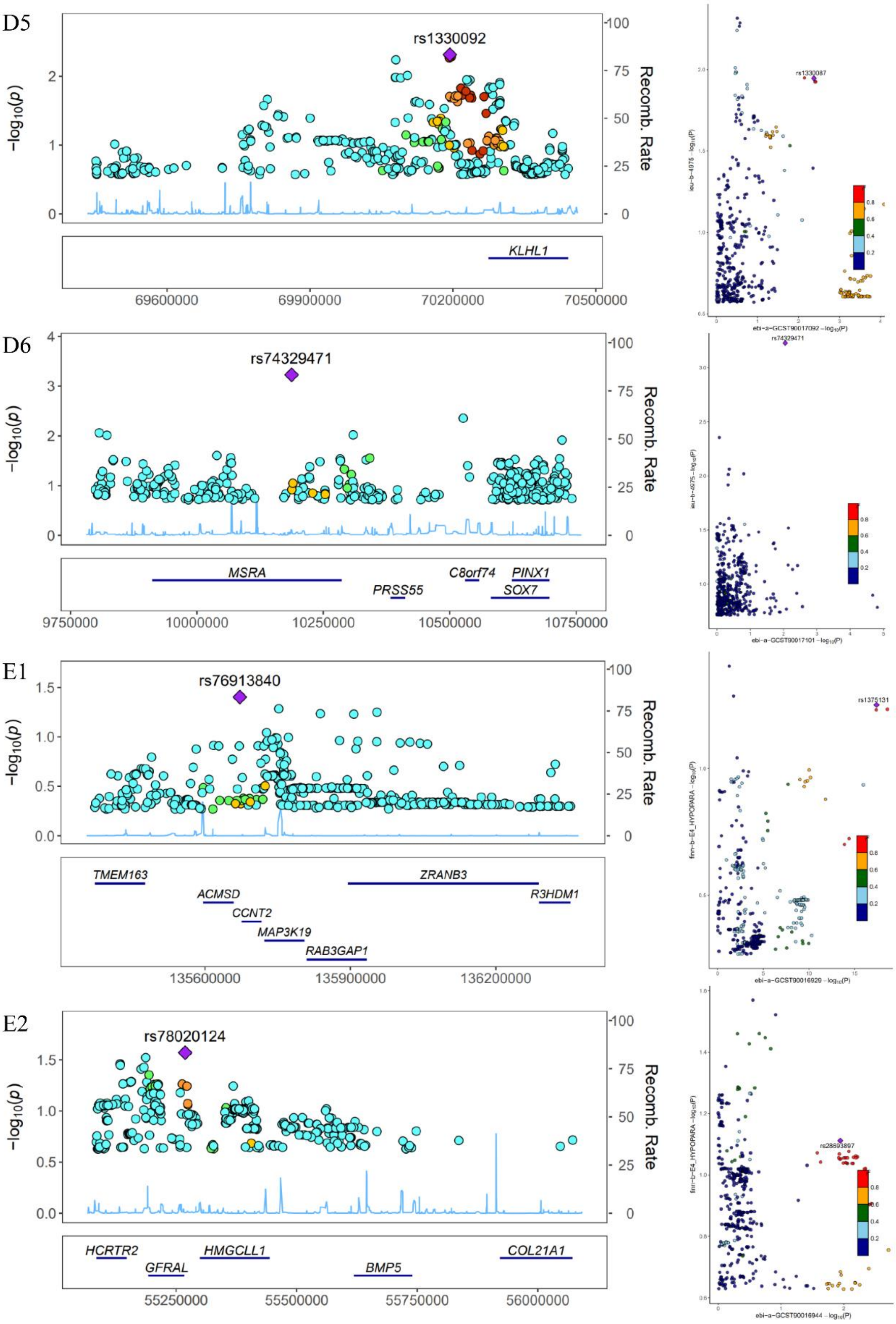
D5
rs1330092
−log10(p)
Recomb. Rate
KLHL1
69600000
69900000
70200000
70500000
rs1330087
D6
rs74329471
MSRA
PRSS55
C8orf74
PINX1
SOX7
9750000
10000000
10250000
10500000
10750000
E1
rs76913840
TMEM163
ACMSD
CCNT2
MAP3K19
RAB3GAP1
ZRANB3
R3HDM1
135600000
135900000
136200000
E2
rs78020124
HCRTR2
GFRAL
HMGCLL1
BMP5
COL21A1
55250000
55500000
55750000
56000000

E3

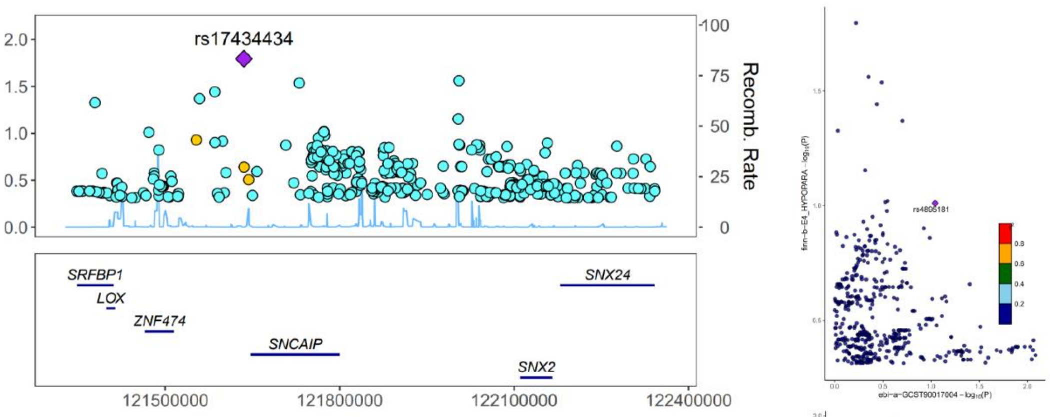
rs17434434
-log10(p)
Recomb. Rate
SRFBP1
LOX
ZNF474
SNCAIP
SNX24
SNX2
121500000
121800000
122100000
122400000
rs4806181
ebi-a-GCST90017004 -log10(P)

E4

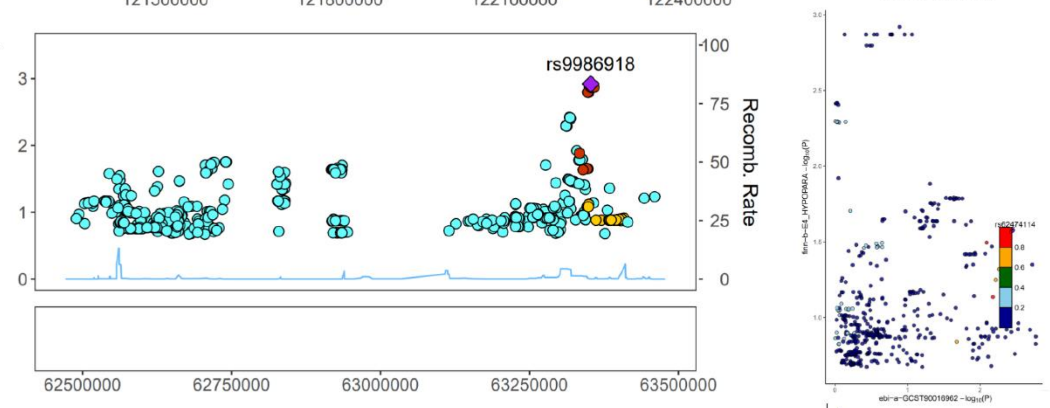
rs9986918
-log10(p)
Recomb. Rate
62500000
62750000
63000000
63250000
63500000
ebi-a-GCST90016962 -log10(P)
finn-b-E4_HYPOPARA -log10(P)

E5

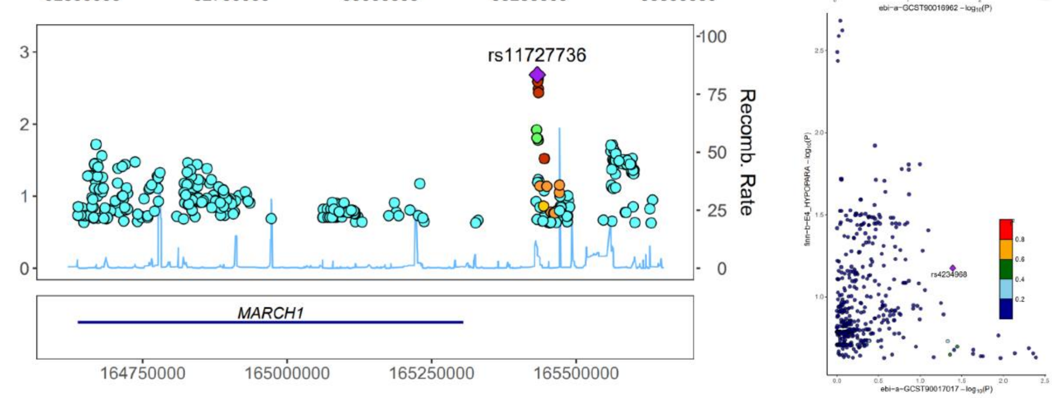
rs11727736
-log10(p)
Recomb. Rate
MARCH1
164750000
165000000
165250000
165500000
rs4234968
ebi-a-GCST90017017 -log10(P)
finn-b-E4_HYPOPARA -log10(P)

E6

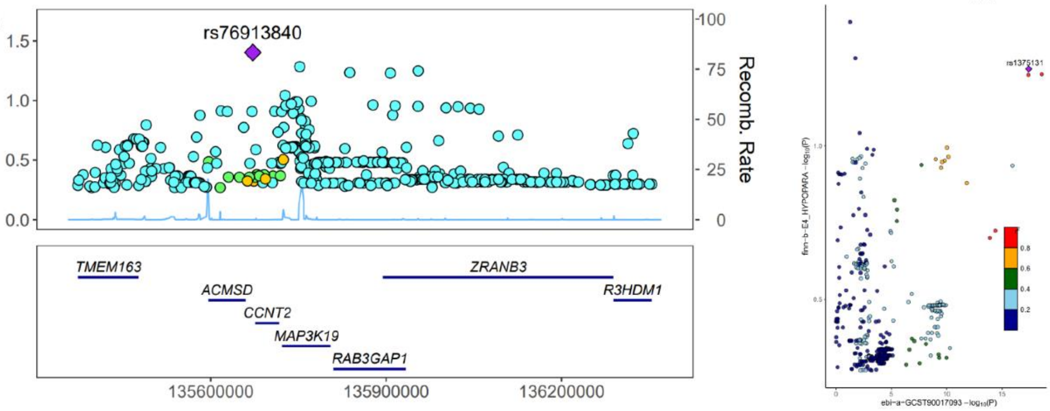
rs76913840
-log10(p)
Recomb. Rate
TMEM163
ACMSD
CCNT2
MAP3K19
RAB3GAP1
ZRANB3
R3HDM1
135600000
135900000
136200000
ebi-a-GCST90017093 -log10(P)
finn-b-E4_HYPOPARA -log10(P)

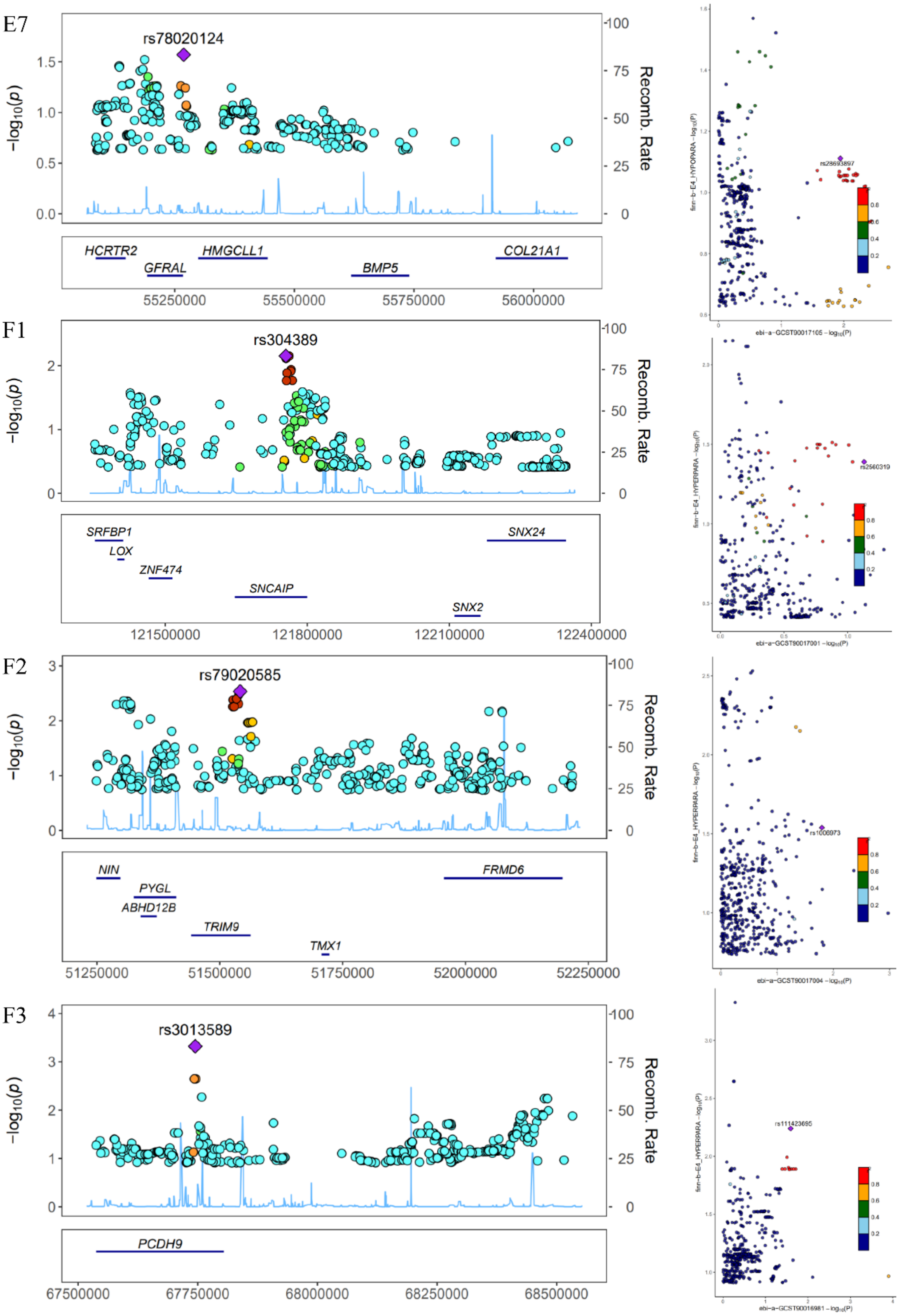
E7
rs78020124
−log10(p)
Recomb. Rate
HCRTR2
GFRAL
HMGCLL1
BMP5
COL21A1
55250000
55500000
55750000
56000000
F1
rs304389
SRFBP1
LOX
ZNF474
SNCAIP
SNX24
SNX2
121500000
121800000
122100000
122400000
F2
rs79020585
NIN
PYGL
ABHD12B
TRIM9
TMX1
FRMD6
51250000
51500000
51750000
52000000
52250000
F3
rs3013589
PCDH9
67500000
67750000
68000000
68250000
68500000

F4

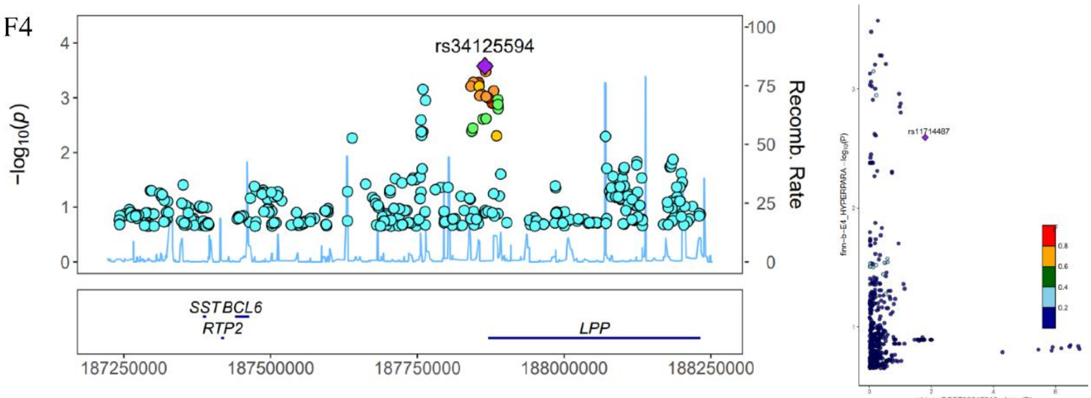

F5

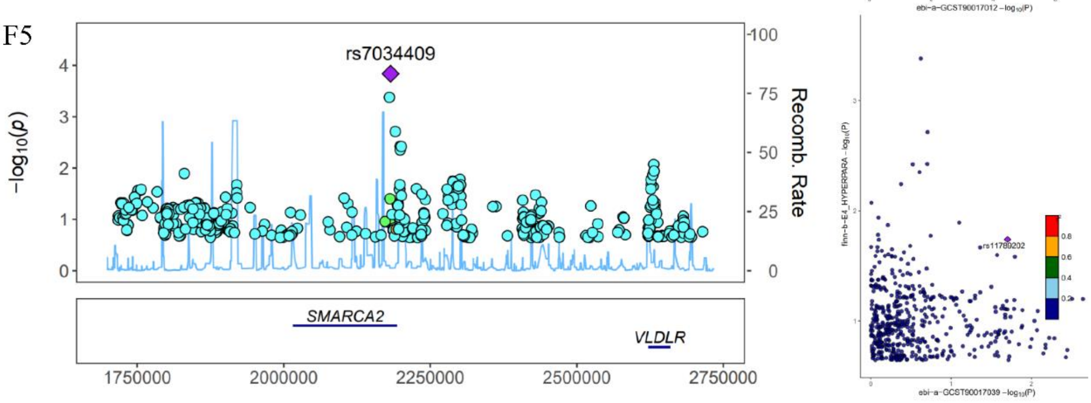

For each colocalized locus (PP.H4 < 0.75) identified for corresponding trait pair, the left panel depicts the PLACO results using LocusZoom plot, and the right panel compares two single-trait GWAS statistics of corresponding trait pair for each variant using LocusCompare plot. For the LocusZoom plot, the x-axis shows the genomic position for each variant, and the y-axis shows -log10 *P* values from PLCAO results. The top variant with the smallest P_{PLACO} in each locus is indicated in purple diamond. The color of each variant represents its LD relationship with the top variant. For the LocusCompare plot, each dot represents a variant, the x-axis shows the -log10 P_{GWAS} from corresponding GWAS of metabolic bone diseases, and the y-axis shows -log10 P_{GWAS} from corresponding GWAS gut microbiota. The candidate shared causal variant identified by pairwise colocalization analysis is also indicated in purple diamond. The color of each variant represents its LD relationship with the candidate shared causal variant. All genomic location is based on reference genome hg19, and LD calculation is based on 1000 Genomes Project of European population. {(A1)genus..Eubacteriumoxidoreducens;(A2)genus.ChristensenellaceaeR.7;(A3)genus.Coprococcus3;(A4)genus.Howardella; (A5) genus.LachnospiraceaeNK4A136} for osteoporosis.

{(B1)genus..Clostridiuminnocuum;(B2)genus.Butyrivibrio;(B3)genus.RuminococcaceaeUCG011;(B4)phylum.Proteobacteria} for Osteopenia.

{(C1)class.Erysipelotrichia;(C2family.Erysipelotrichaceae;(C3)family.FamilyXIII;(C4)genus.ChristensenellaceaeR.7;(C5)genus.Dorea;(C6)genus.Parabacteroides;(C7)order.Erysipelotrichales} for Osteonecrosis.

{(D11)class.Bacilli;(D2)class.Bacteroidia;(D3)family.BacteroidalesS24.7;(D4)genus.Butyricimonas;(NA)genus.Lachnospira;(D5)order.Bacteroidales; (D6)

order.Lactobacillales} for Osteomyelitis.

{(E1)family.Bifidobacteriaceae;(E2)family.Pasteurellaceae;(E3)genus..Eubacteriumruminantium;(E4)genus.Alistipes;(E5)genus.Hungatella;(E6)order.Bifidobacteriales;(E7)order.Pasteurellales} for Hypoparathyroidism.

{(F1)genus..Eubacteriumnodatum;(F2)genus..Eubacteriumruminantium;(F3)genus.Collinsella;(F4)genus.Gordonibacter;(F5)genus.Parabacteroides} for Hyperparathyroidism
